# Supplementary material for: Perinatal Ethanol Exposure Induces Astrogliosis and Decreases GRP55/PEA-Mediated Neuroprotection in Hippocampal Astrocytes of the 3×Tg Alzheimer’s Animal Model
Source: Int J Mol Sci. 2025 Nov 18;26(22):11154. doi: 10.3390/ijms262211154 (PMC12652644; doi:10.3390/ijms262211154)
Supplement: Supplementary file 1 [file ijms-26-11154-s001.zip › Figure S1. Unedited blots.pdf]

Western Blot Results
Perinatal 3xTg EtOH Astrocytes

ibima
Instituto de Investigación
Biomédica de Málaga

3xTg astrocytes

CB1 (53 kDa)
CB2 (38-40 kDa)

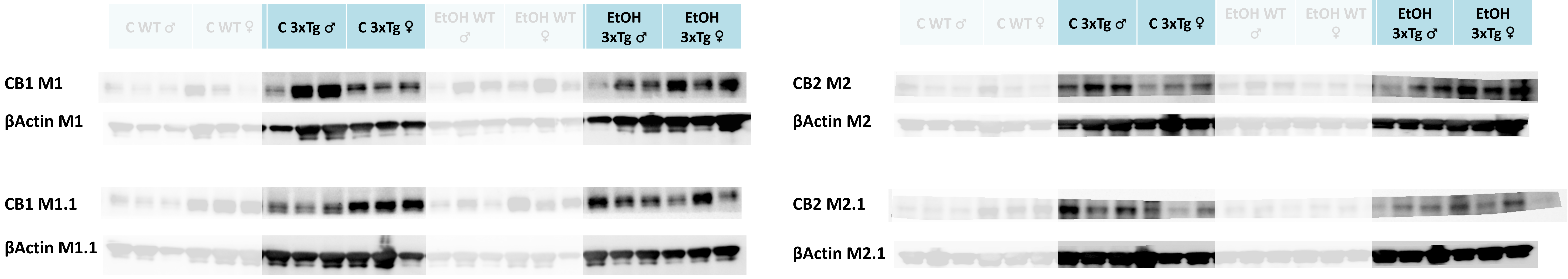

Data for each gel with an n of 3 animals. Both gels have a total n of 6.

PONCEAU RED STAINING

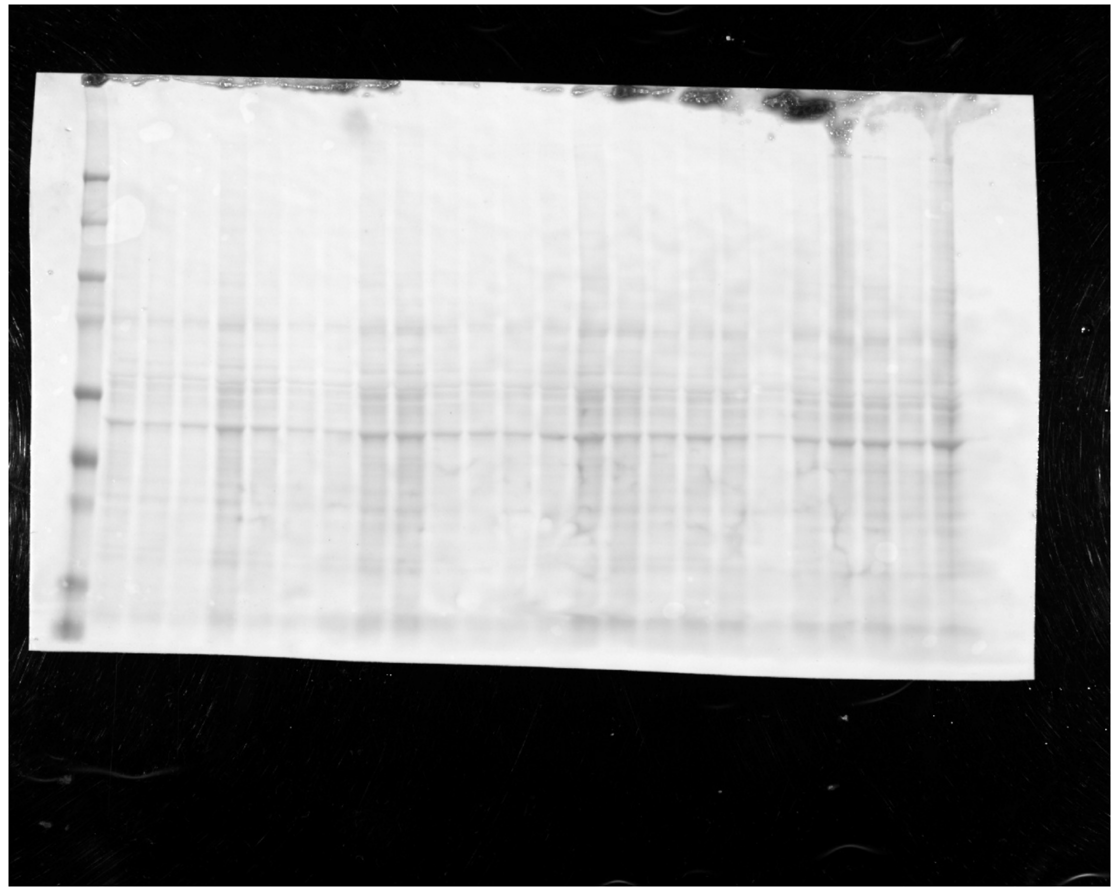

M1

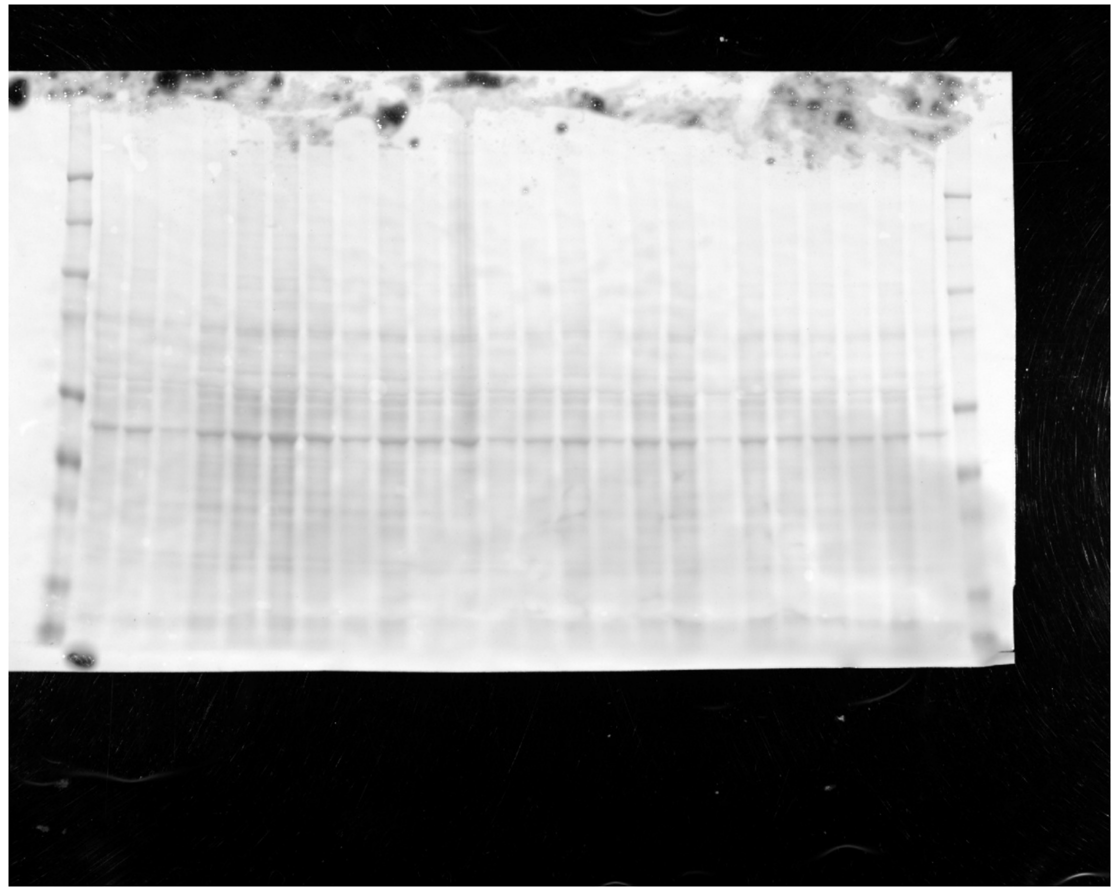

M1.1

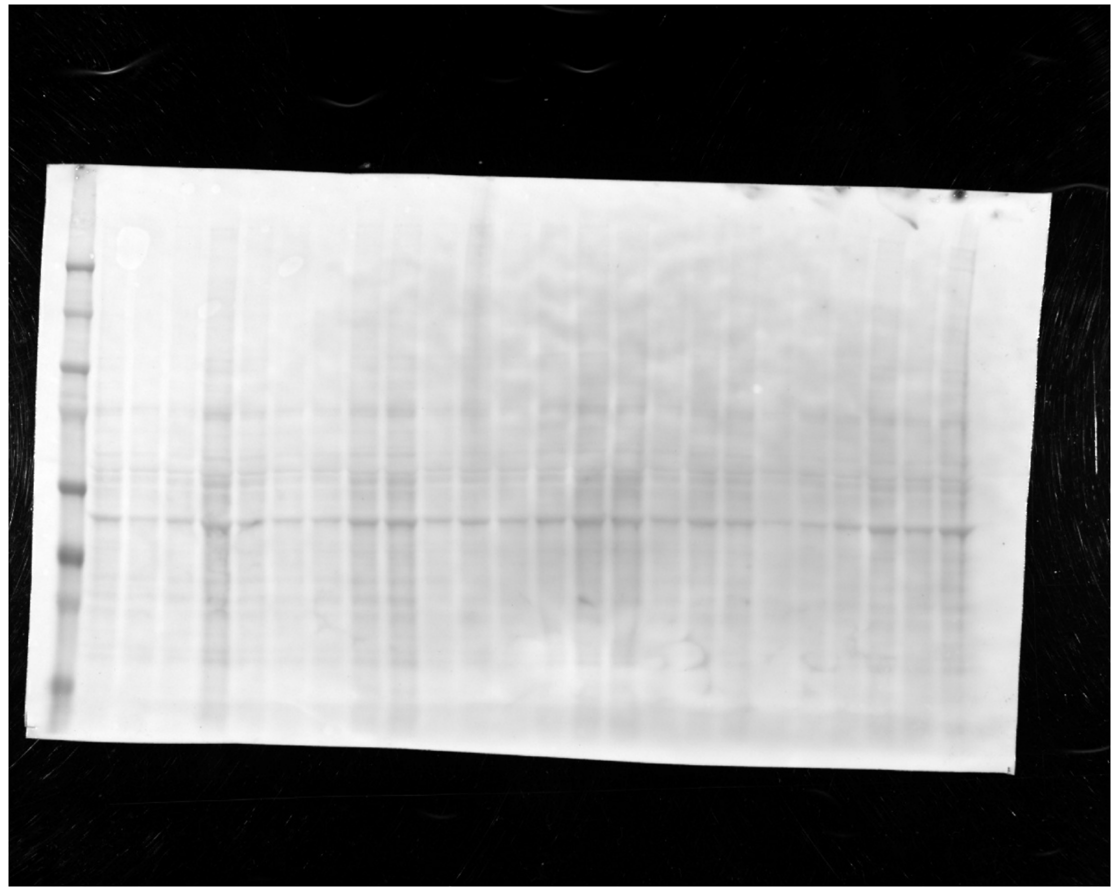

M2

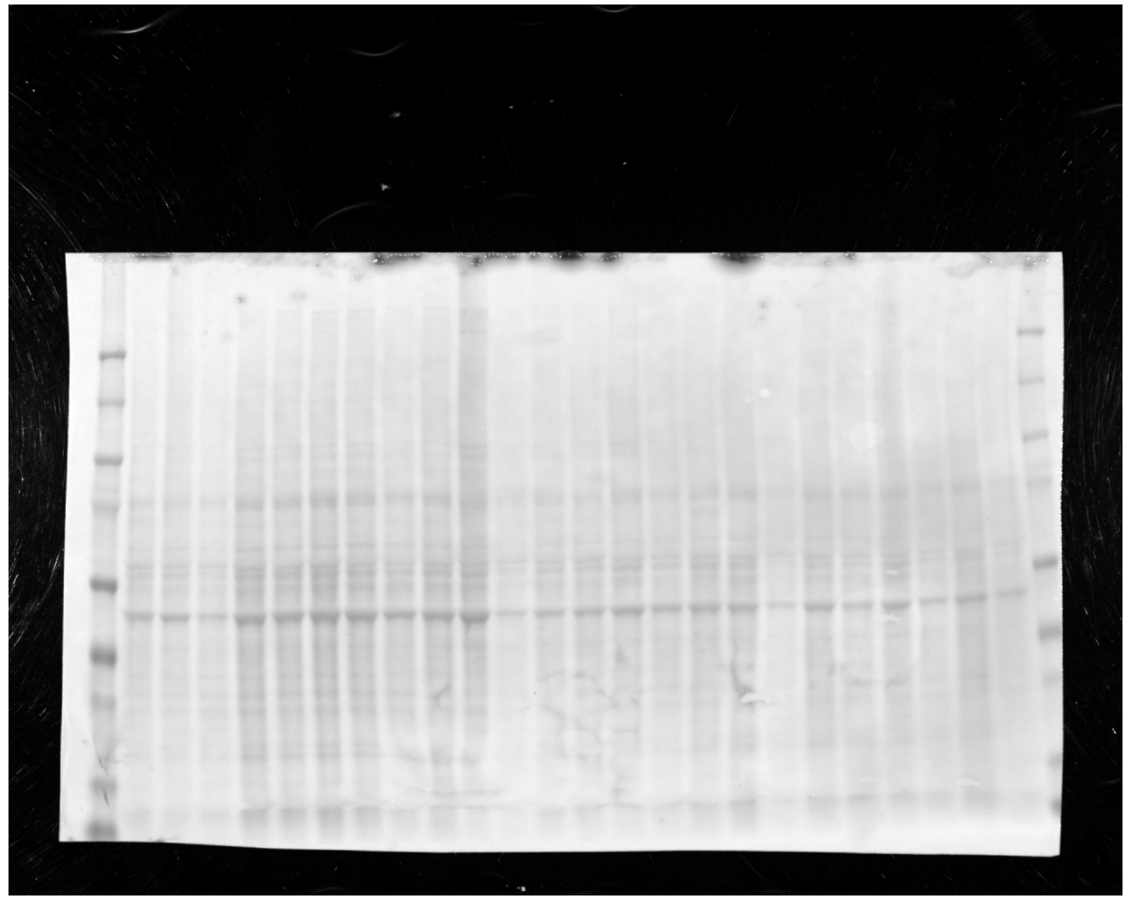

M2.1

UNEDITED BLOTS: βActin

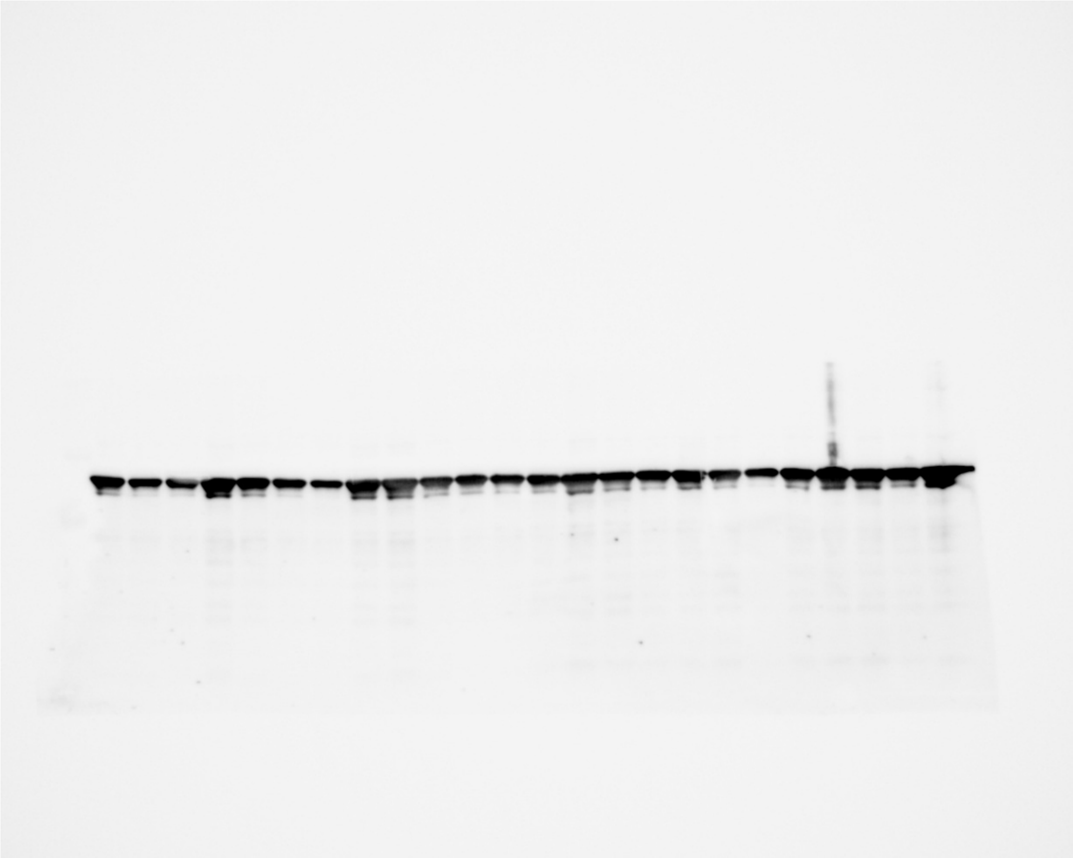

βActin M1

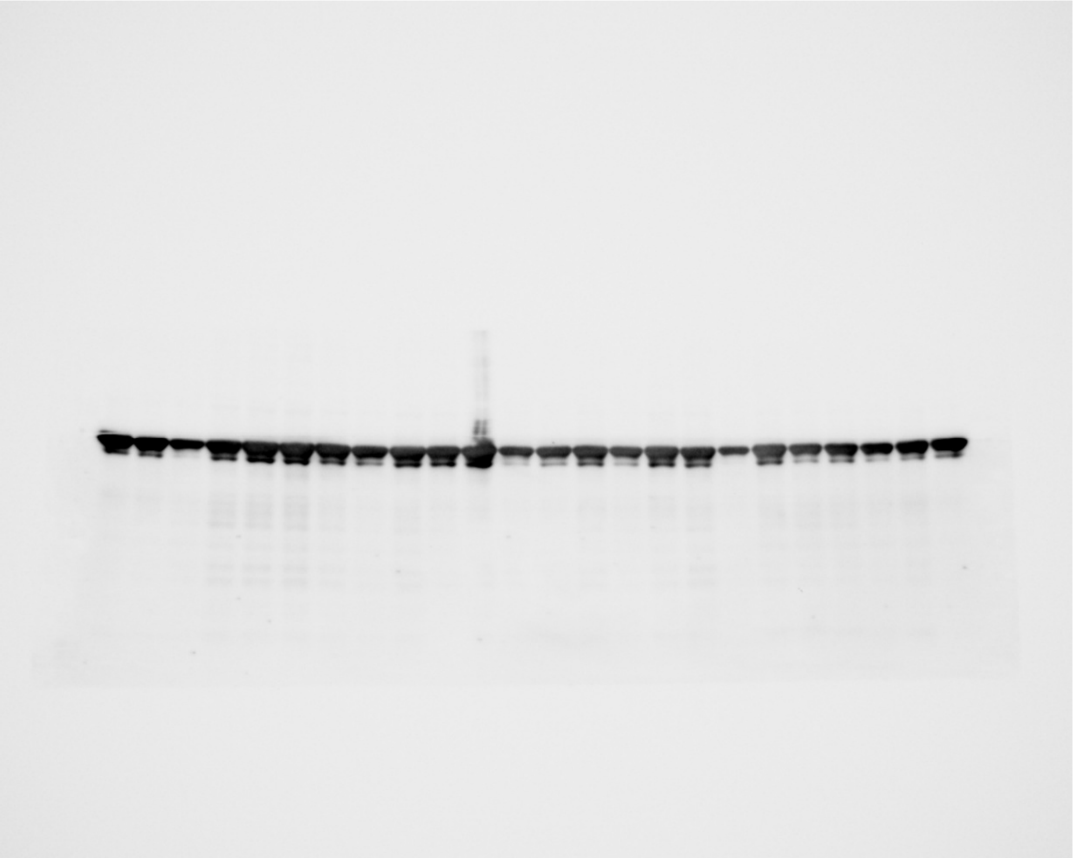

βActin M1.1

UNEDITED BLOTS: CB1 and CB2

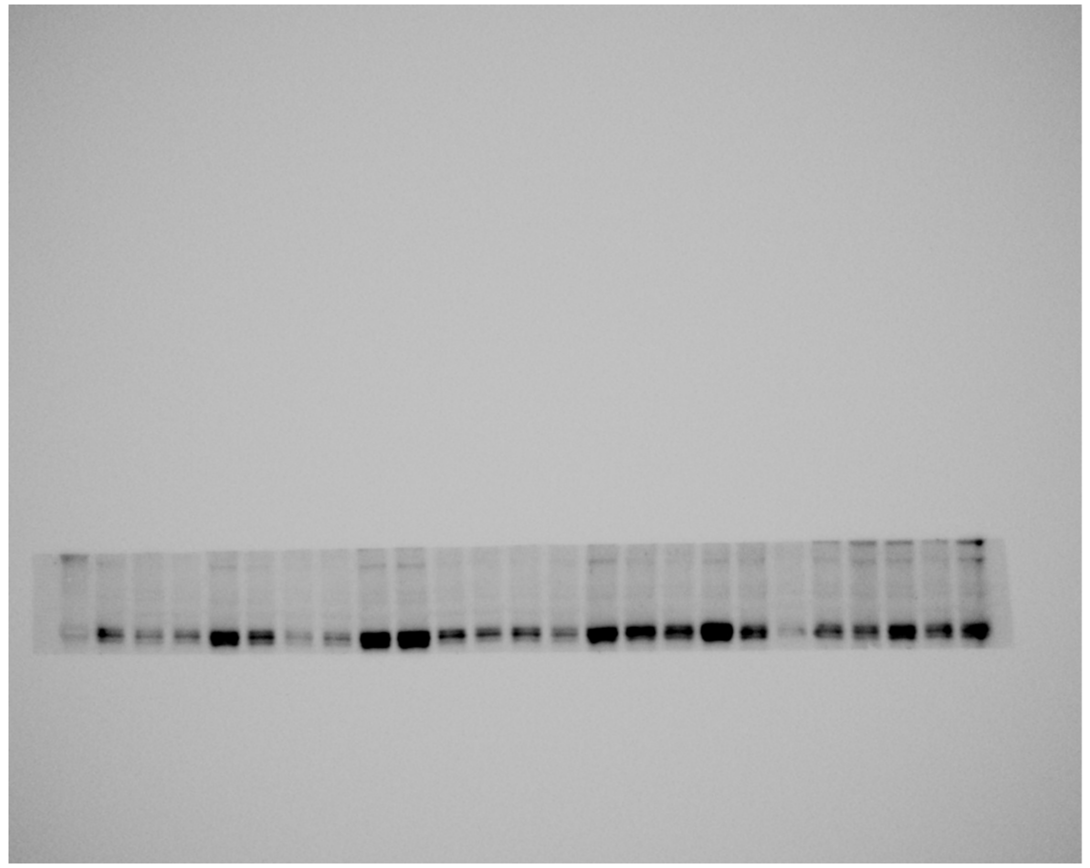

CB1 M1

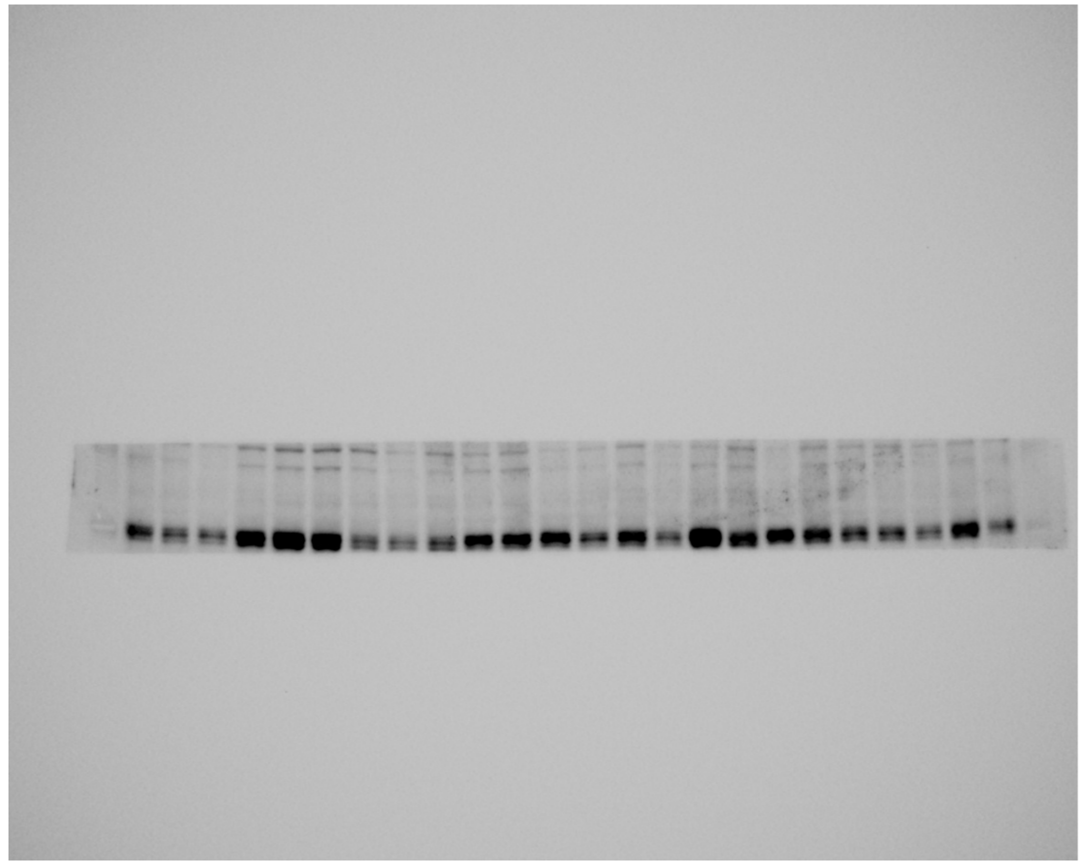

CB1 M1.1

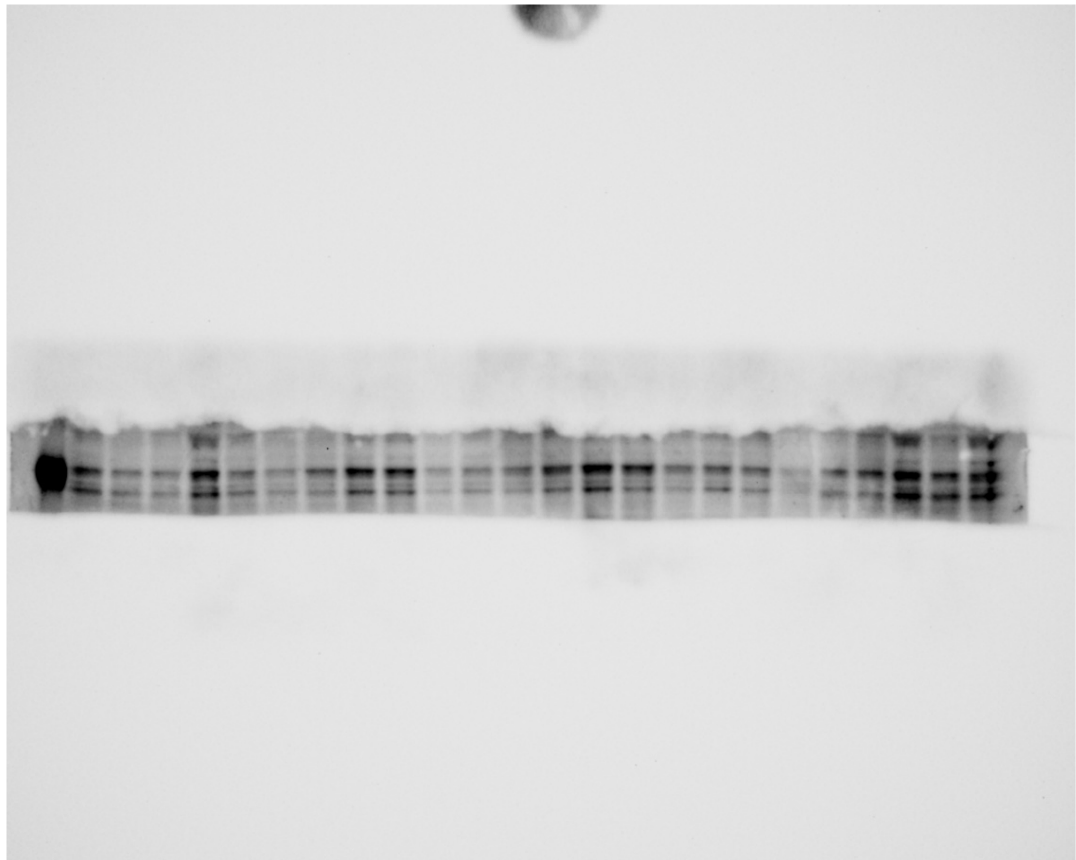

CB2 M2

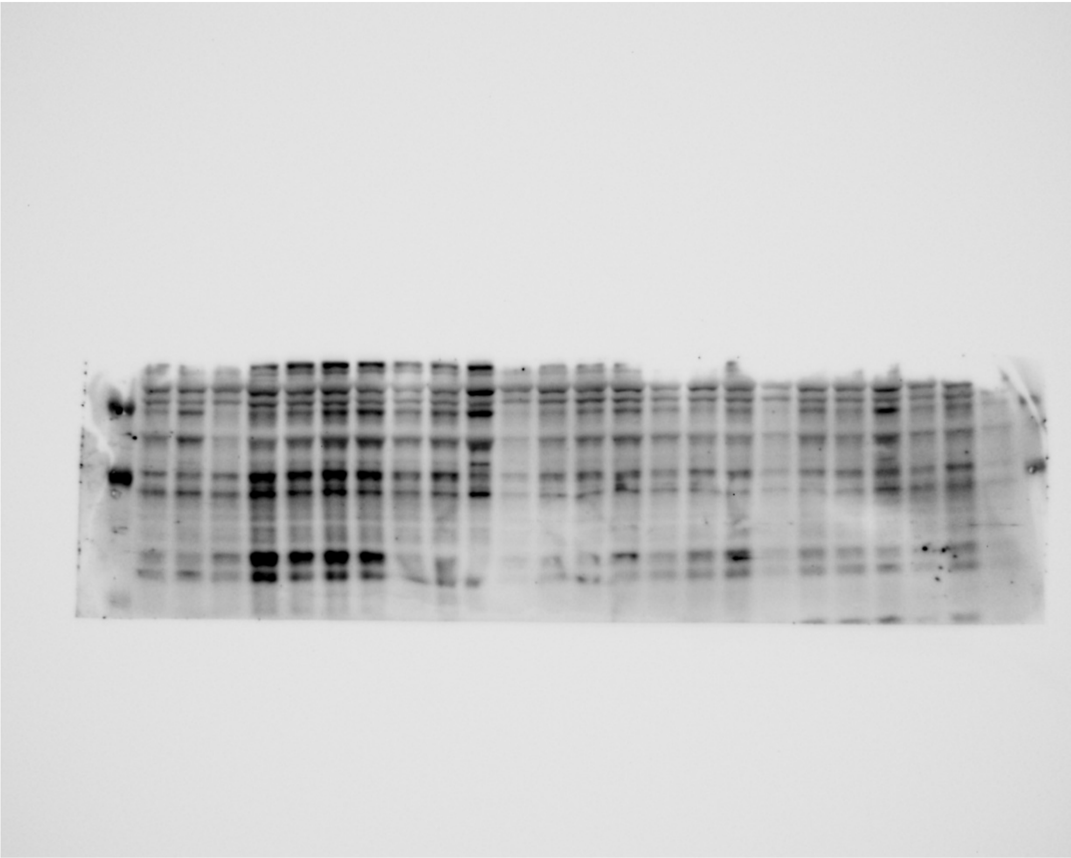

CB2 M2.1

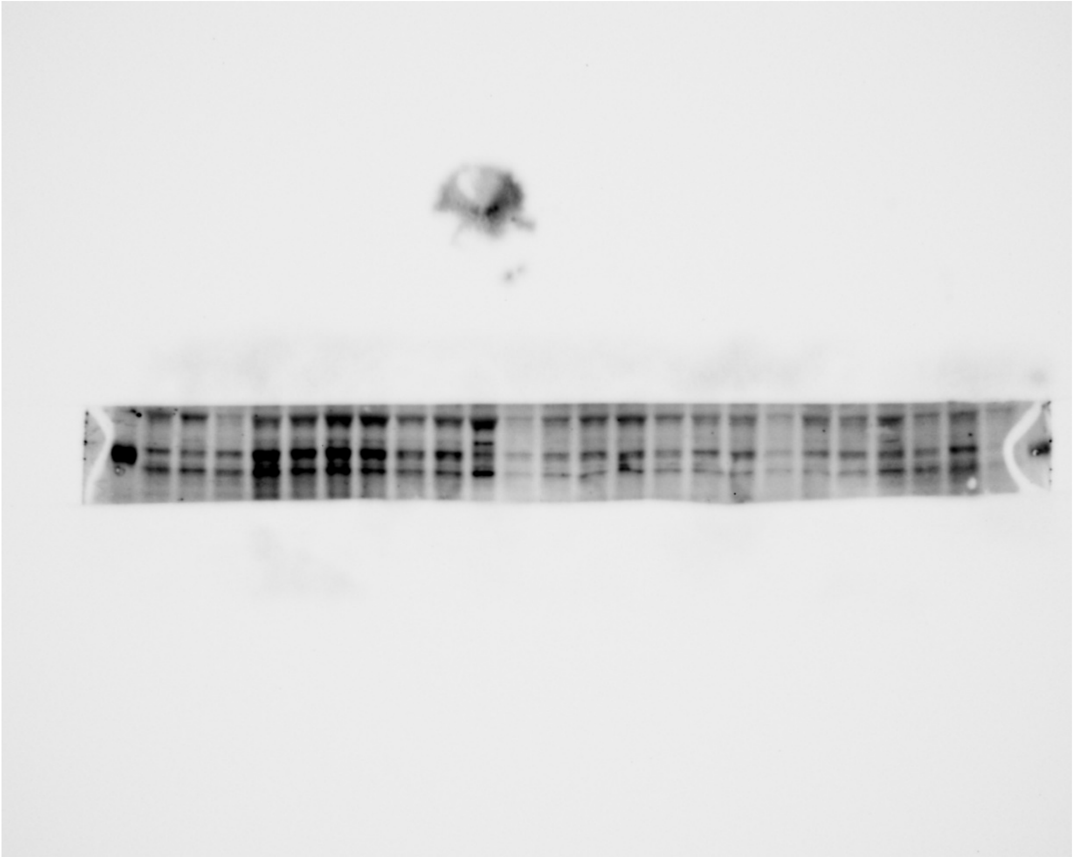

CB2 M2.1

3xTg astrocytes

DAGLα (115 kDa)
DAGLβ (74 kDa)

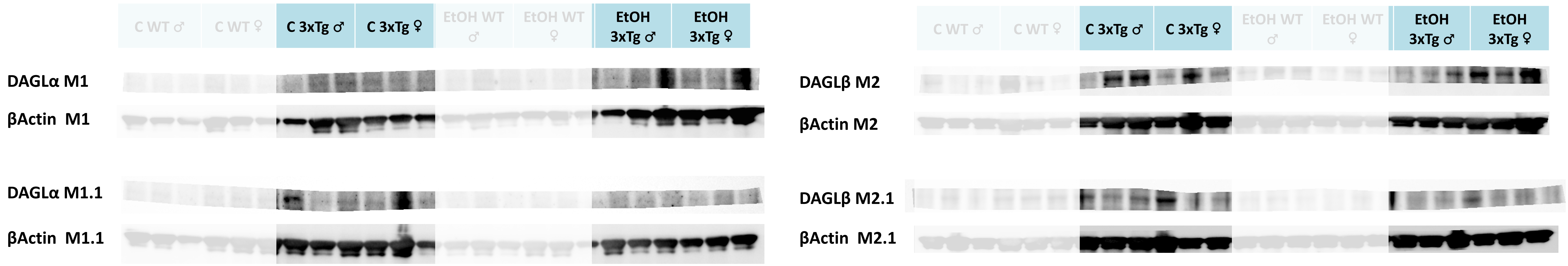

Data for each gel with an n of 3 animals. Both gels have a total n of 6.

UNEDITED BLOTS: DAGLα and DAGLβ

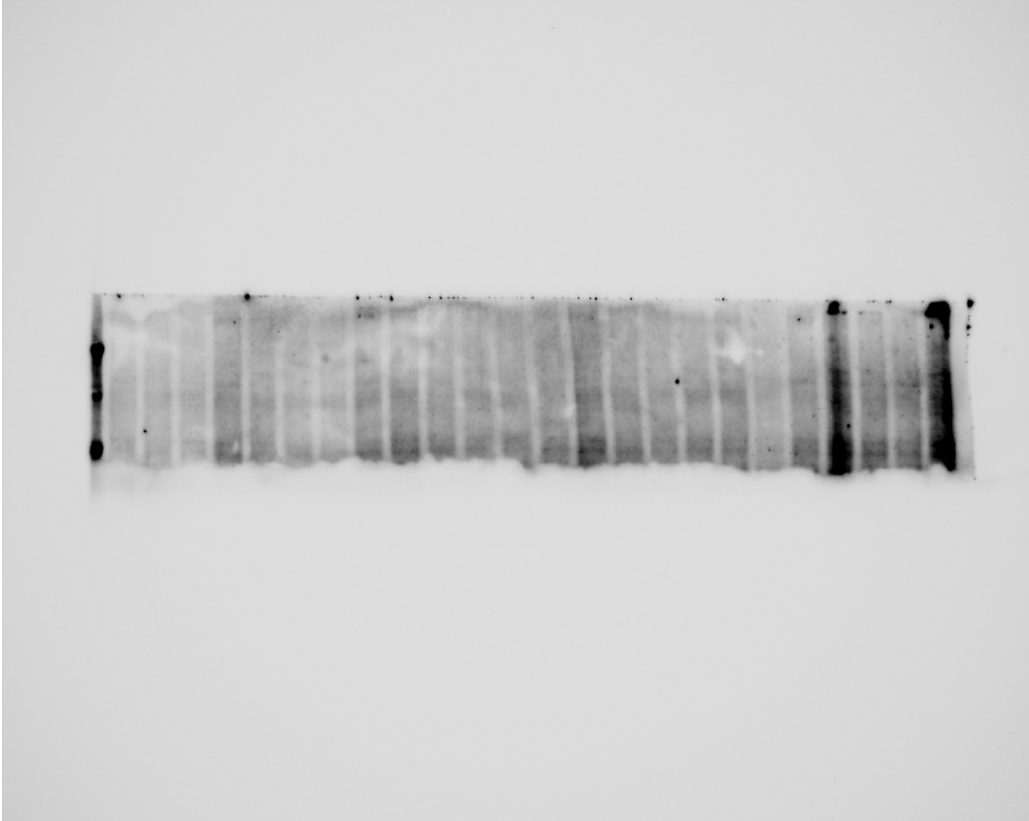

DAGLα M1

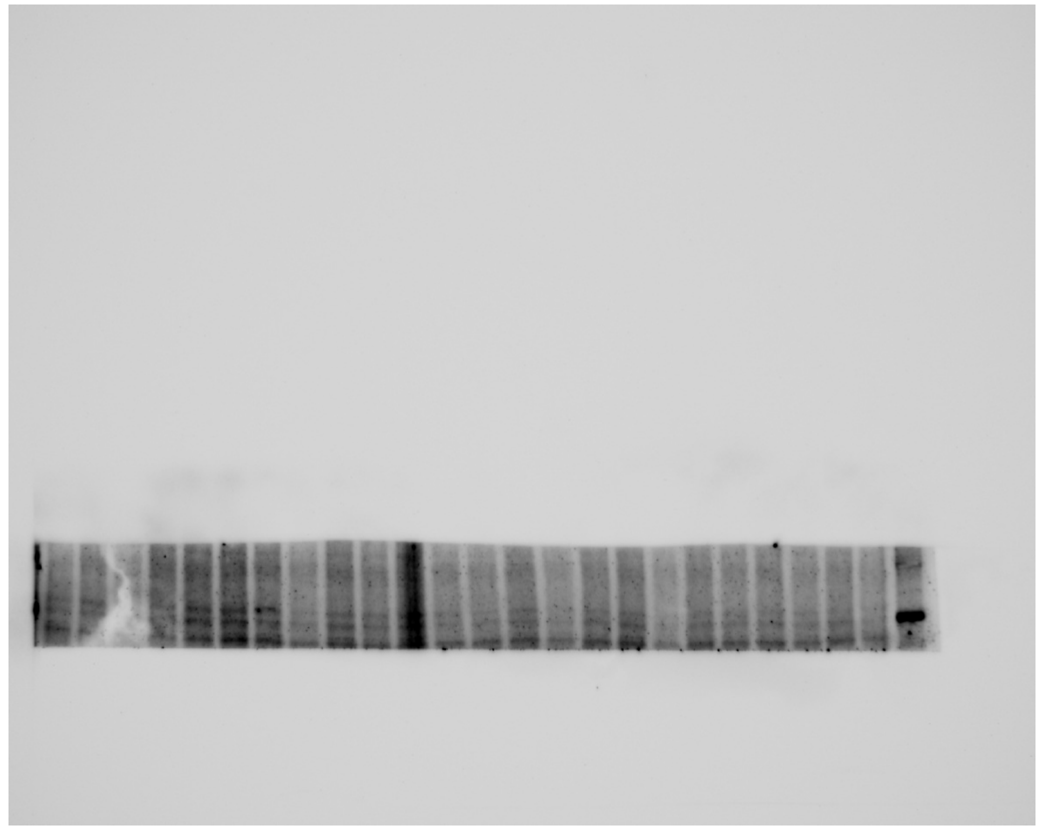

DAGLα M1.1

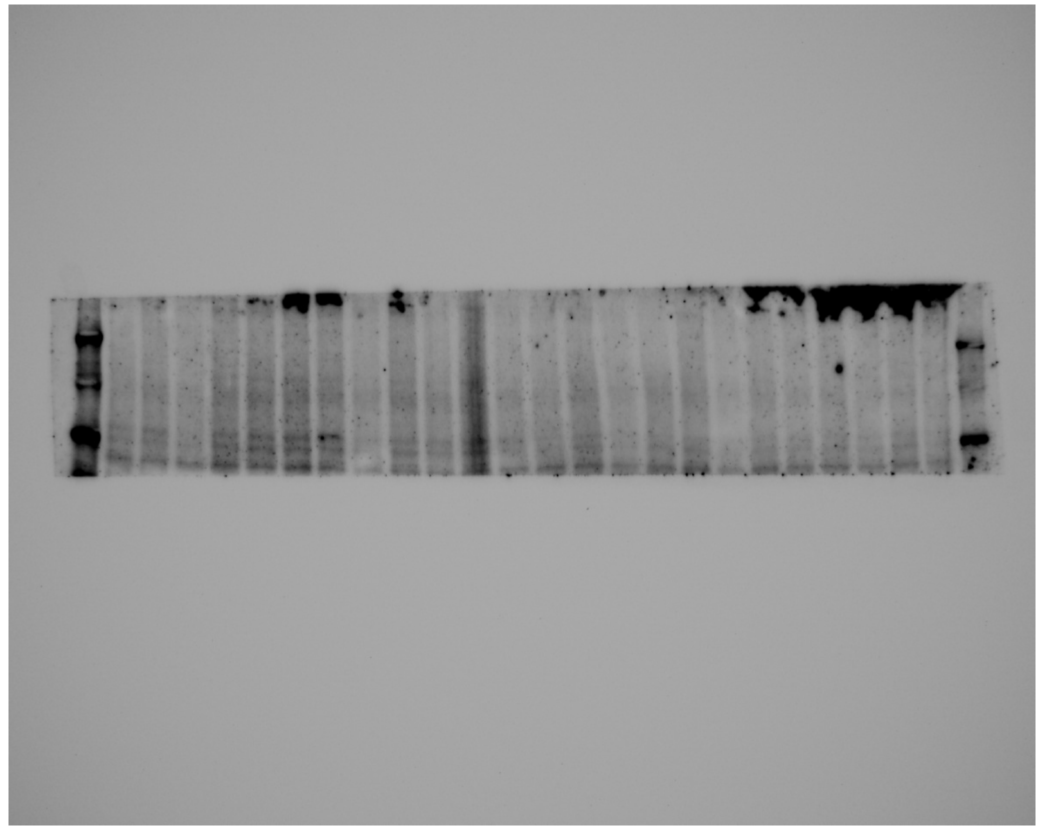

DAGLα M1.1 (complete)

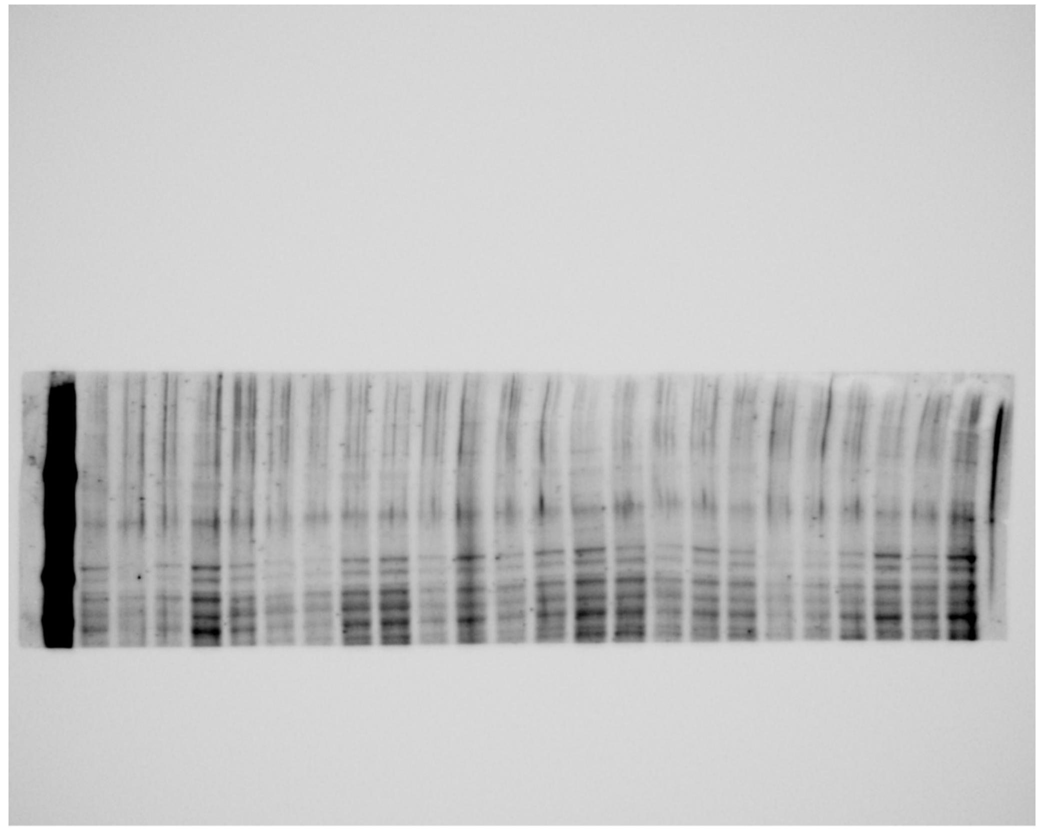

DAGLβ M2

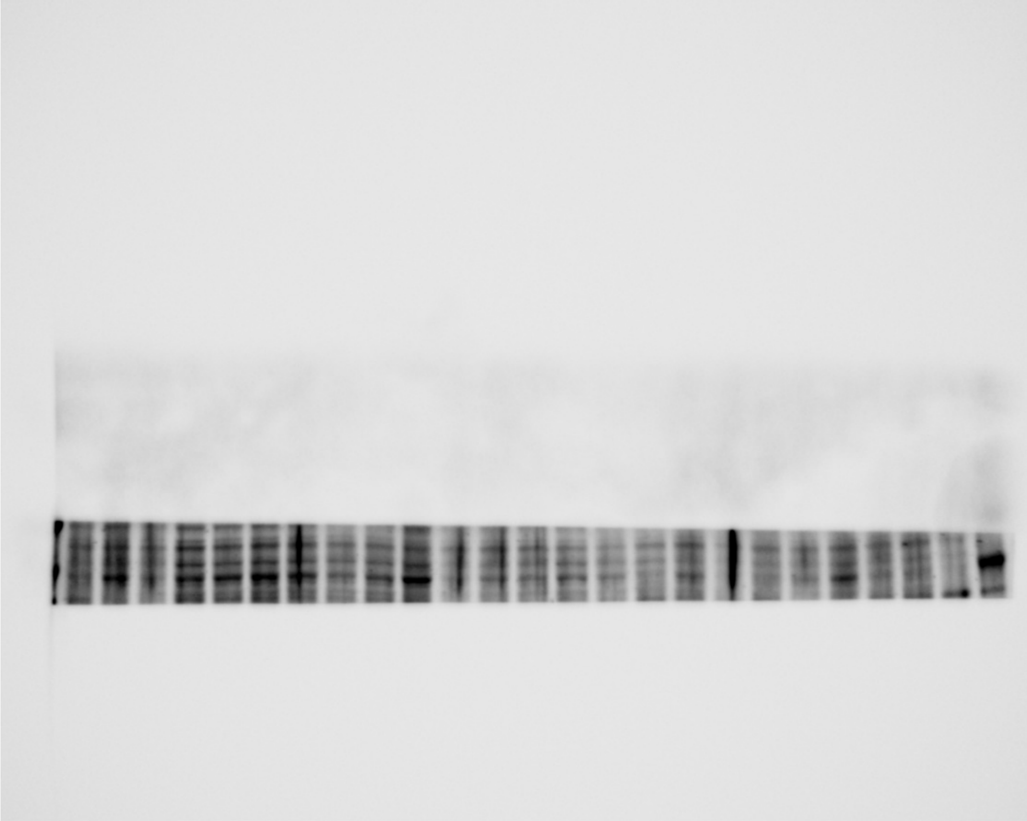

DAGLβ M2.1

3xTg astrocytes

PPARα (53 kDa)

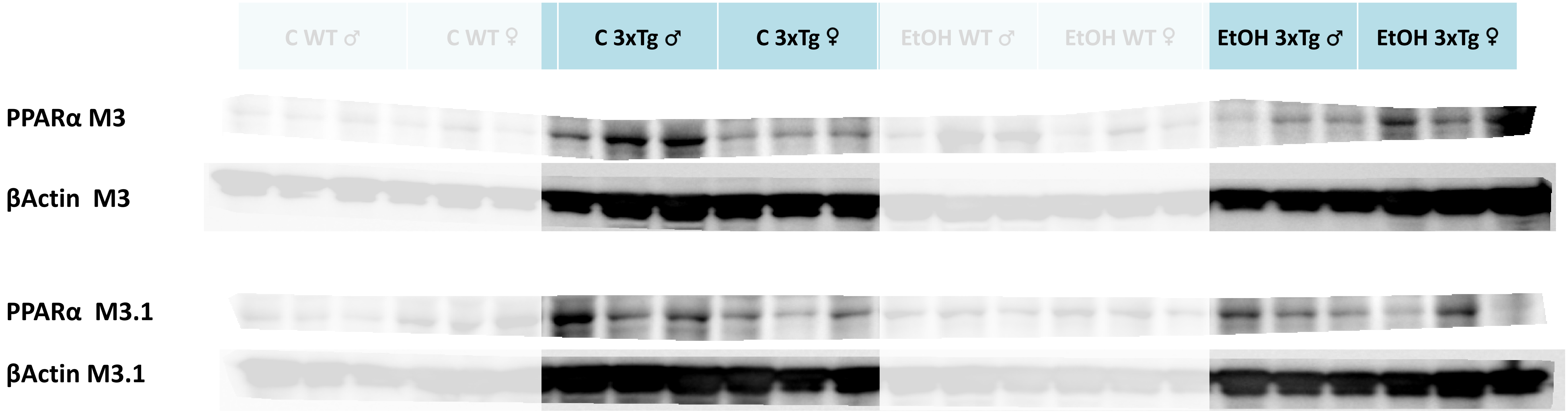

Data for each gel with an n of 3 animals. Both gels have a total n of 6.

PONCEAU RED STAINING

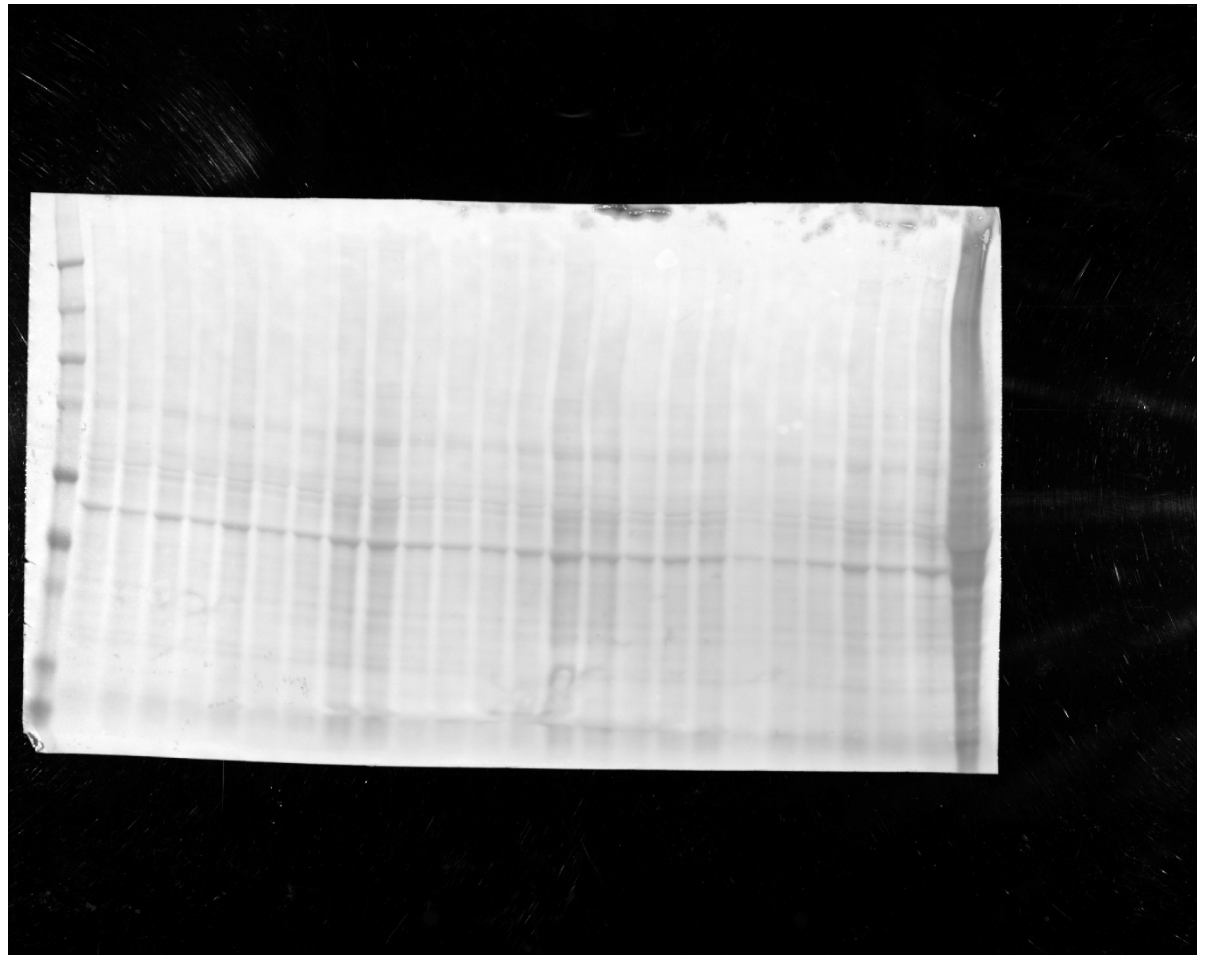

M3

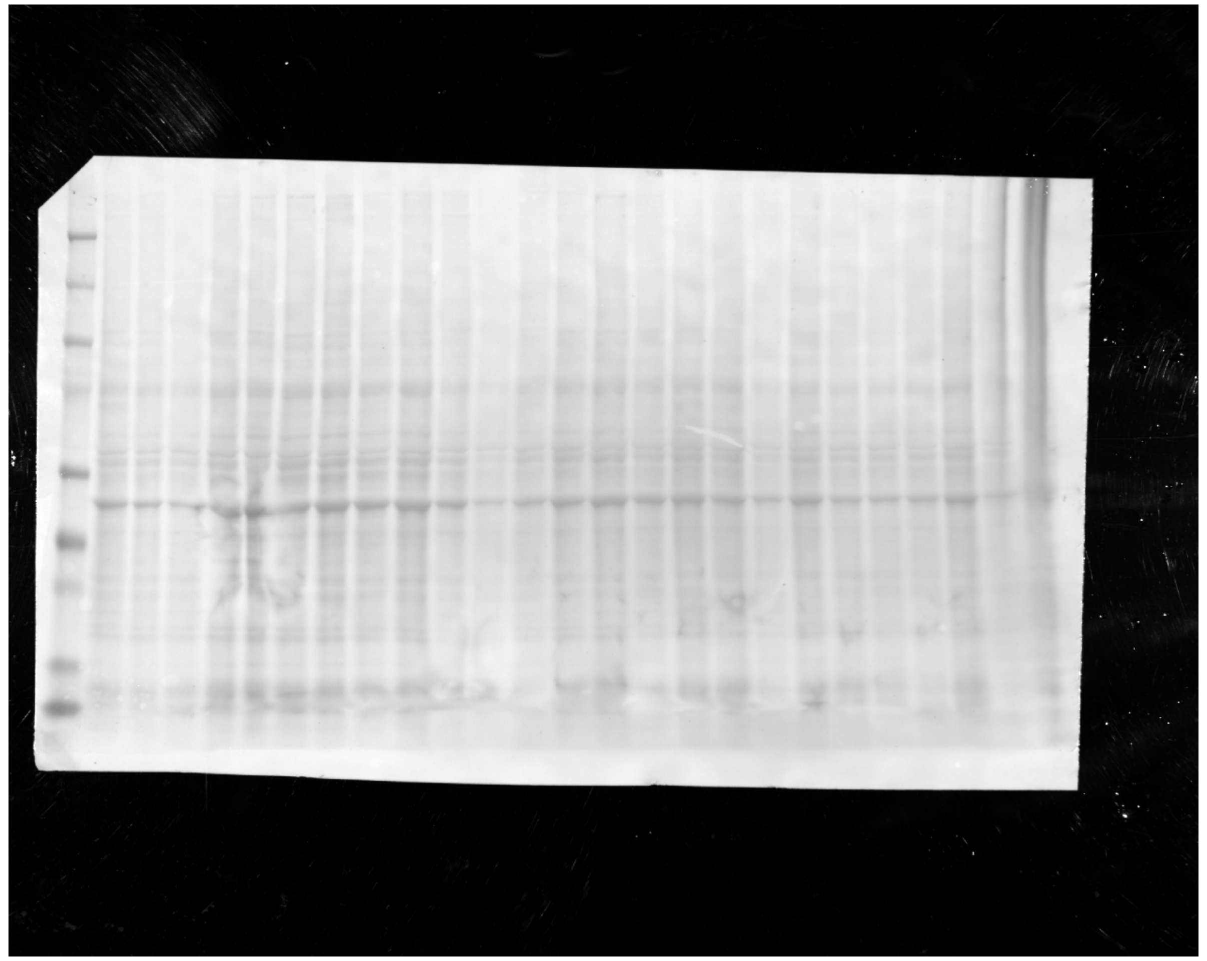

M3.1

UNEDITED BLOTS: βActin

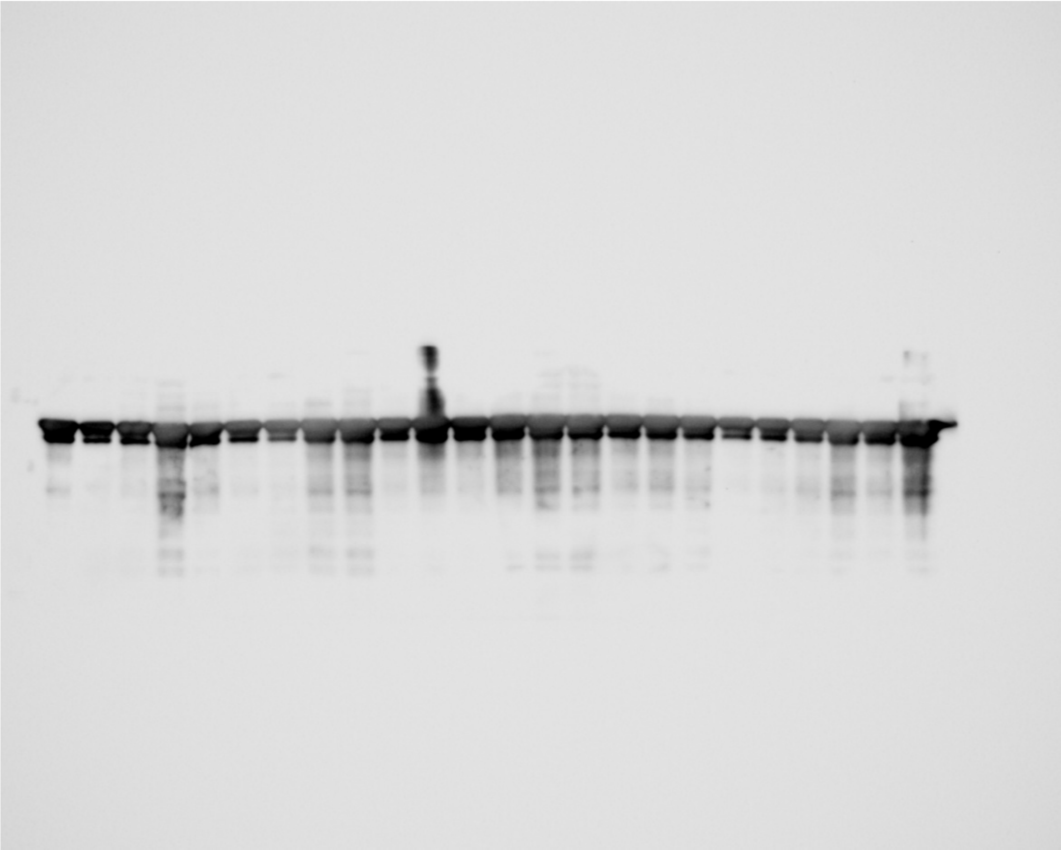

βActin M2

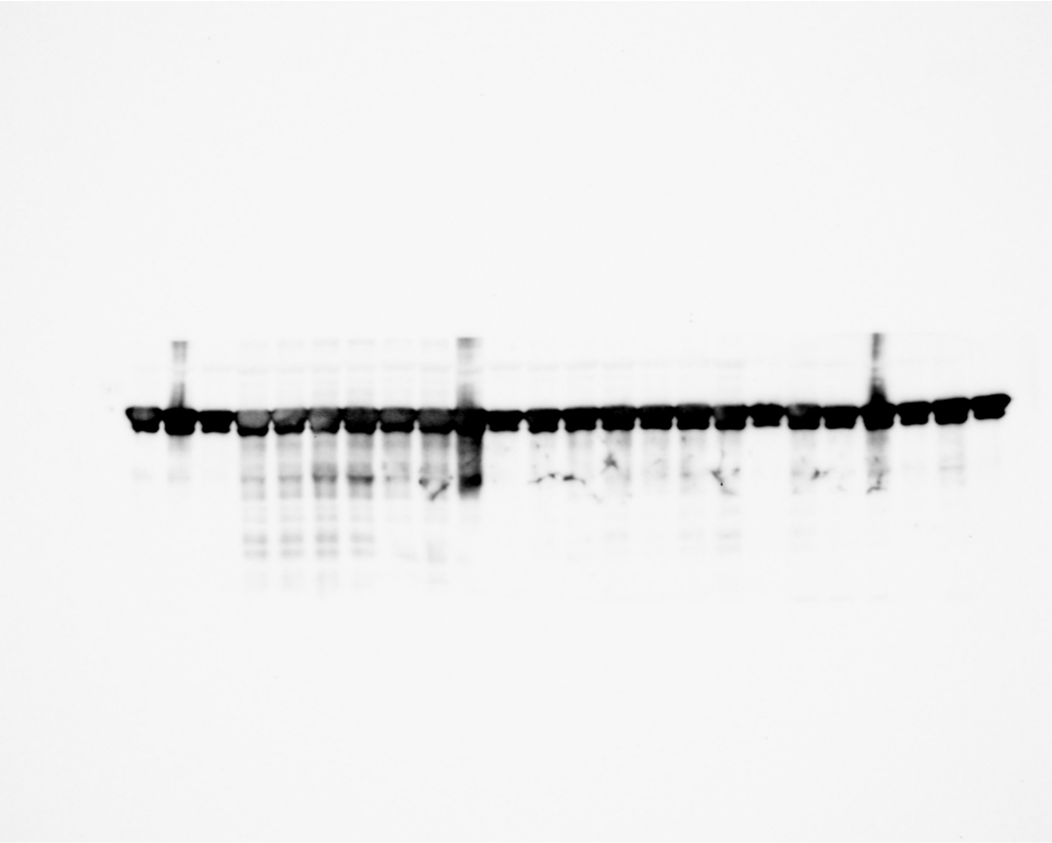

βActin M2.1

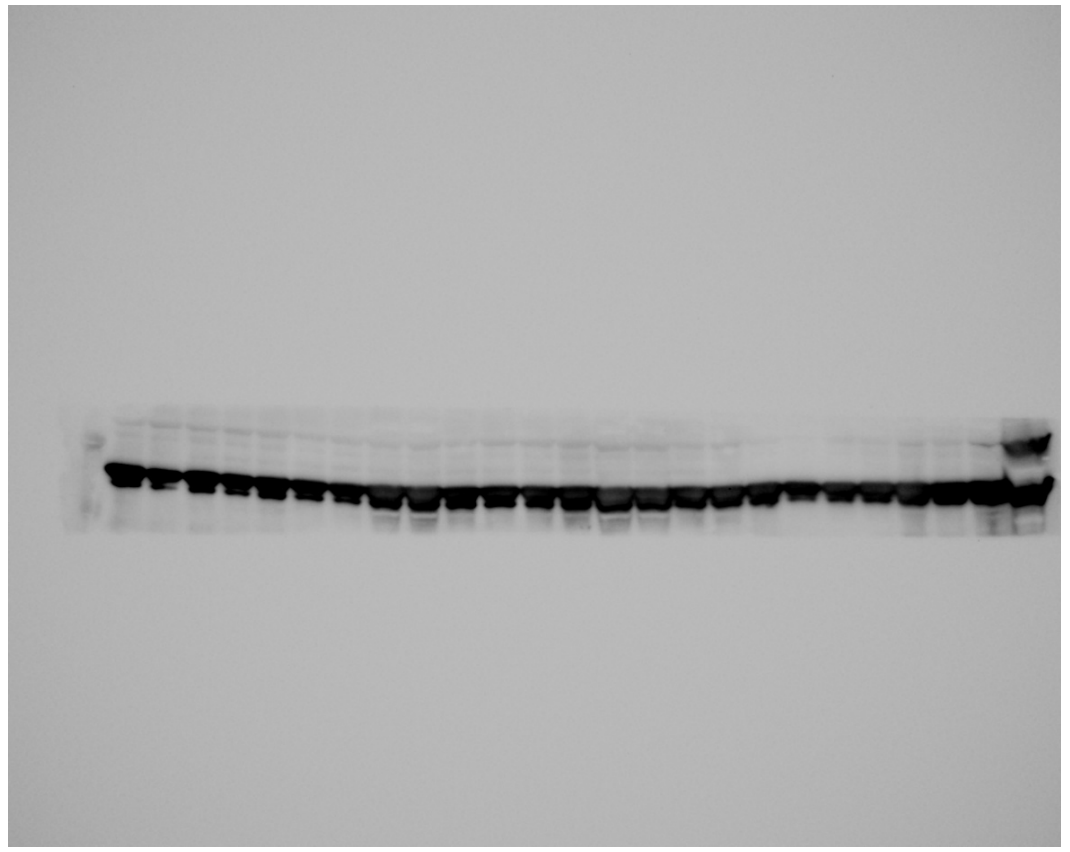

βActin M3

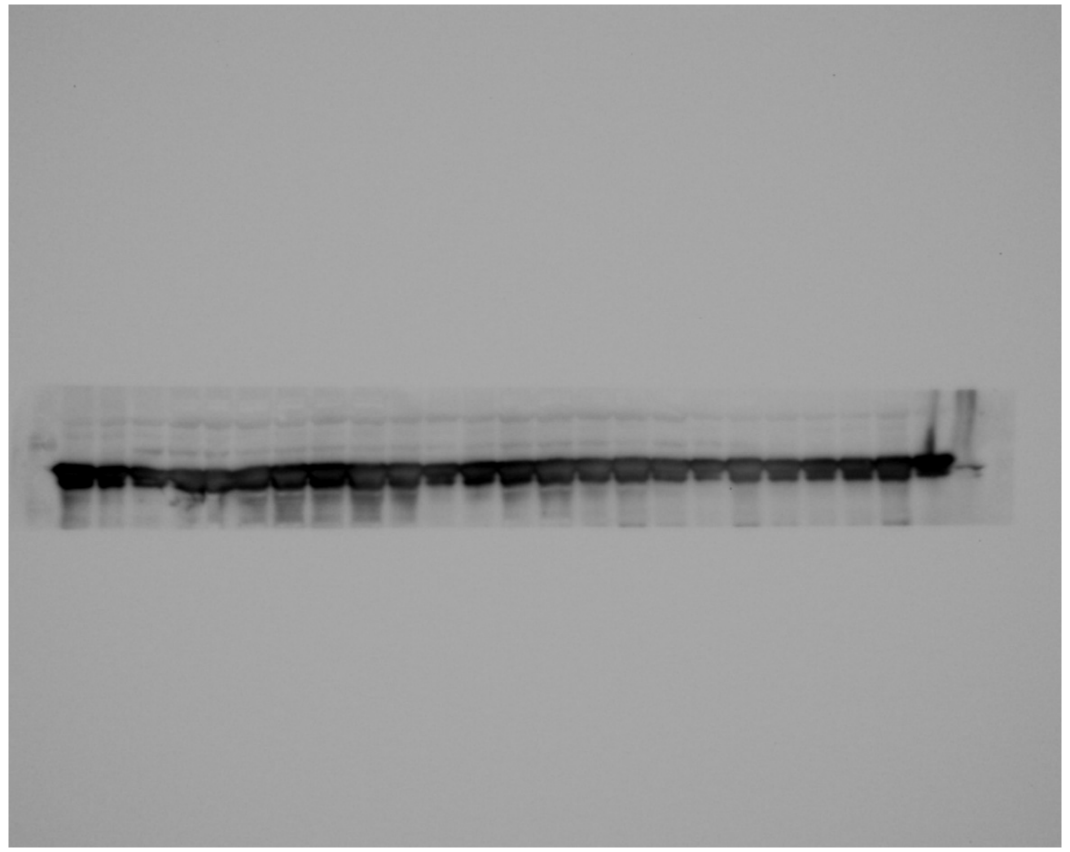

βActin M3.1

UNEDITED BLOTS: PPARα

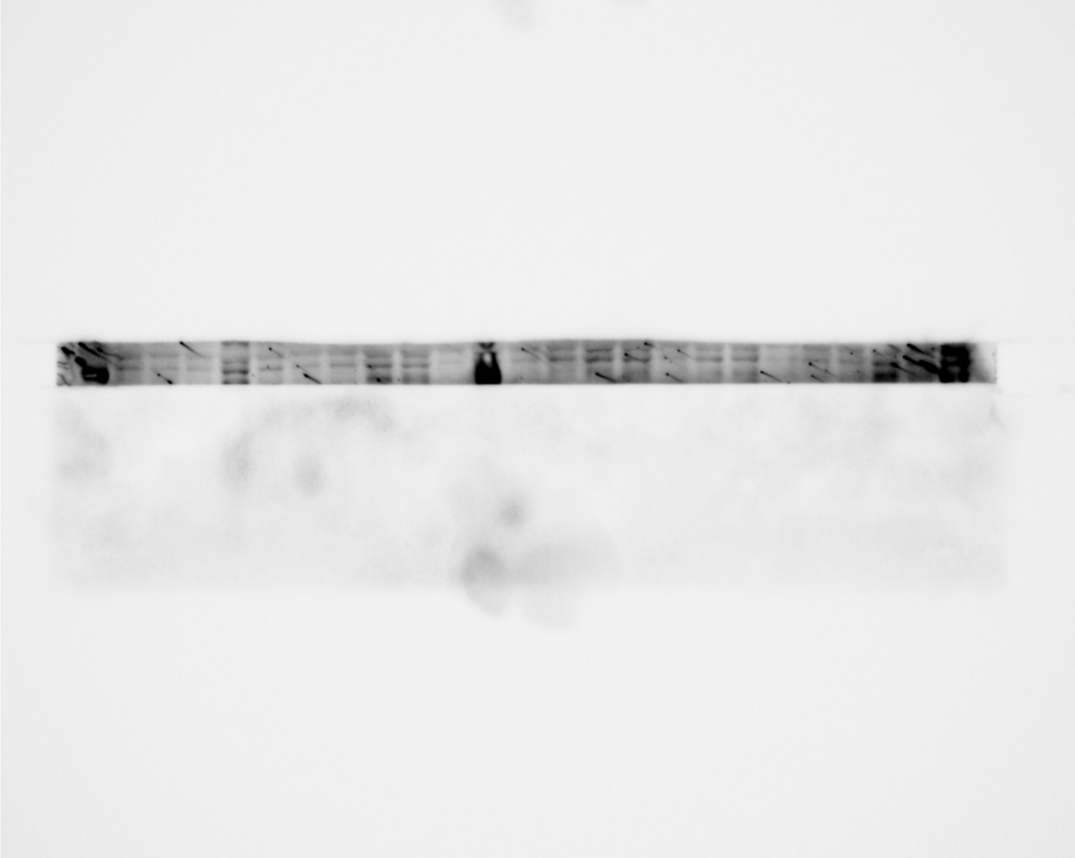

PPARα M2

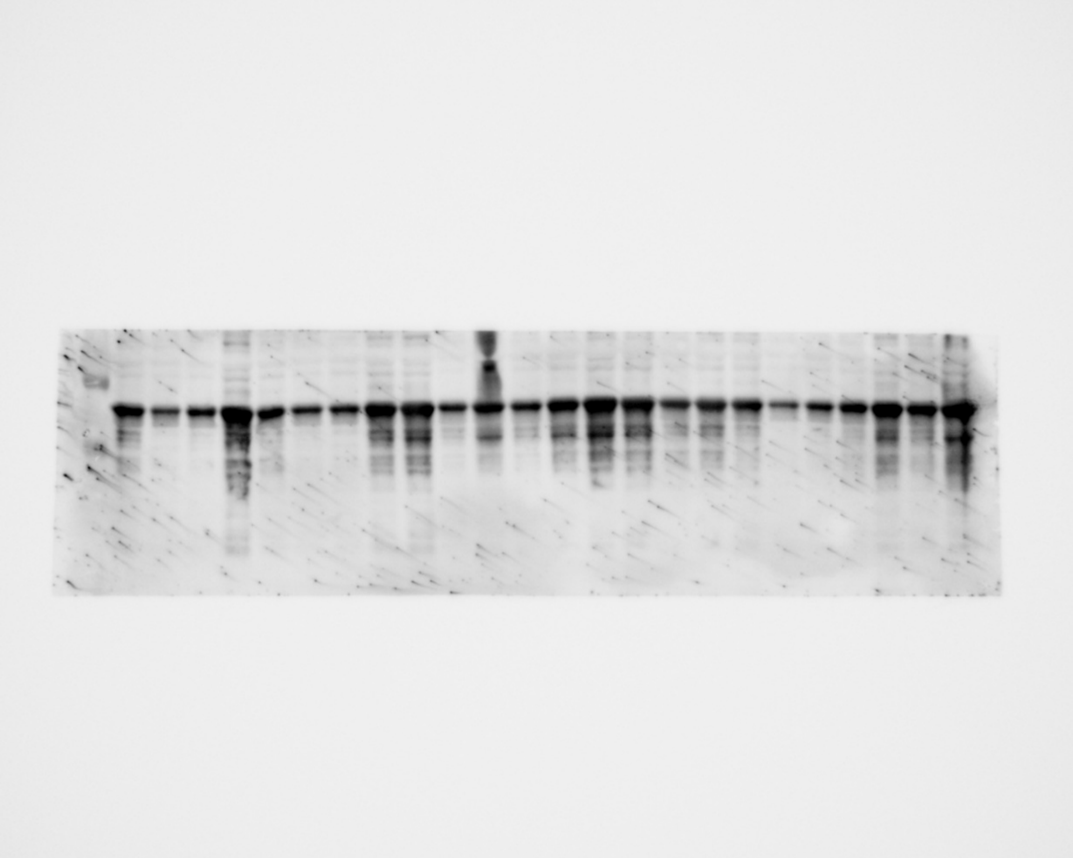

PPARα M2

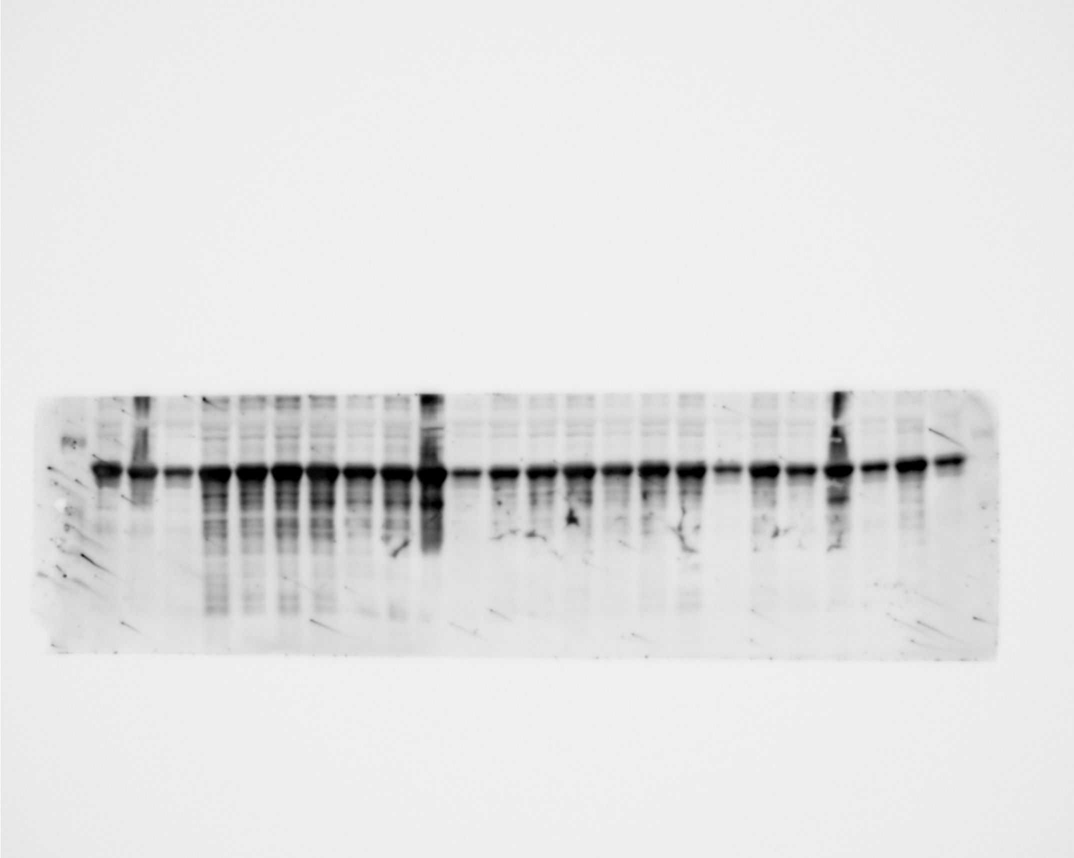

PPARα M2.1

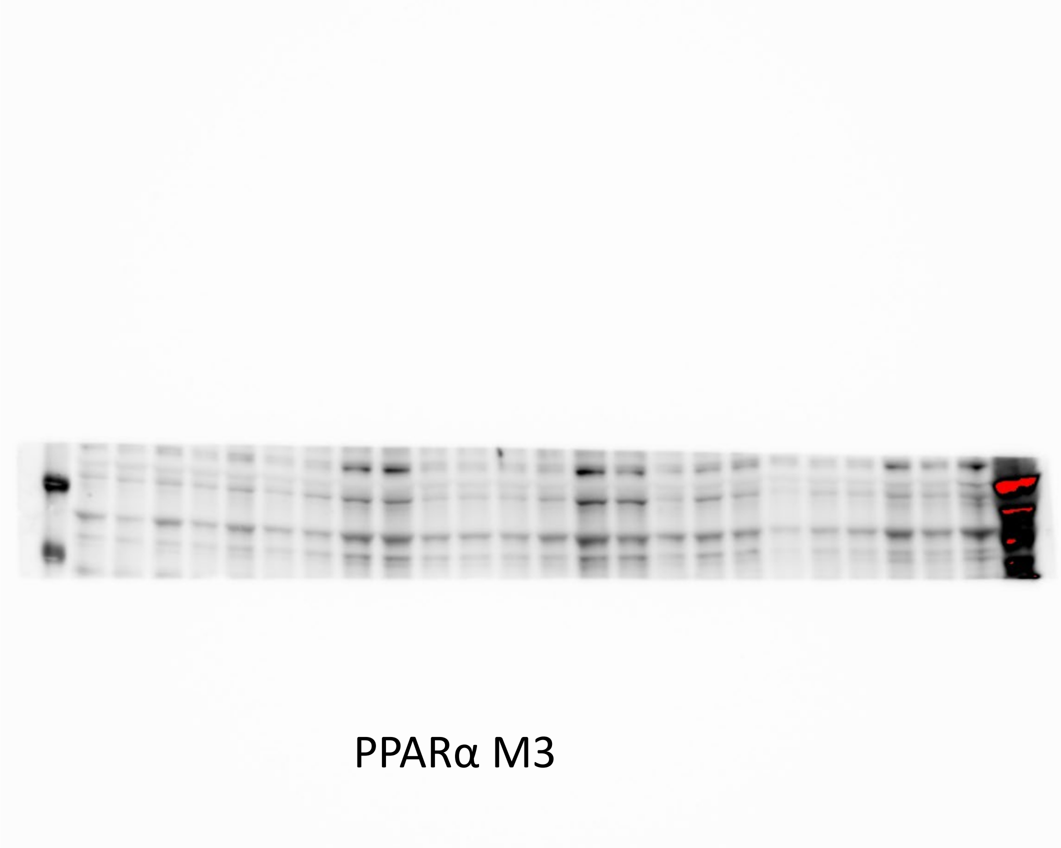

PPARα M3

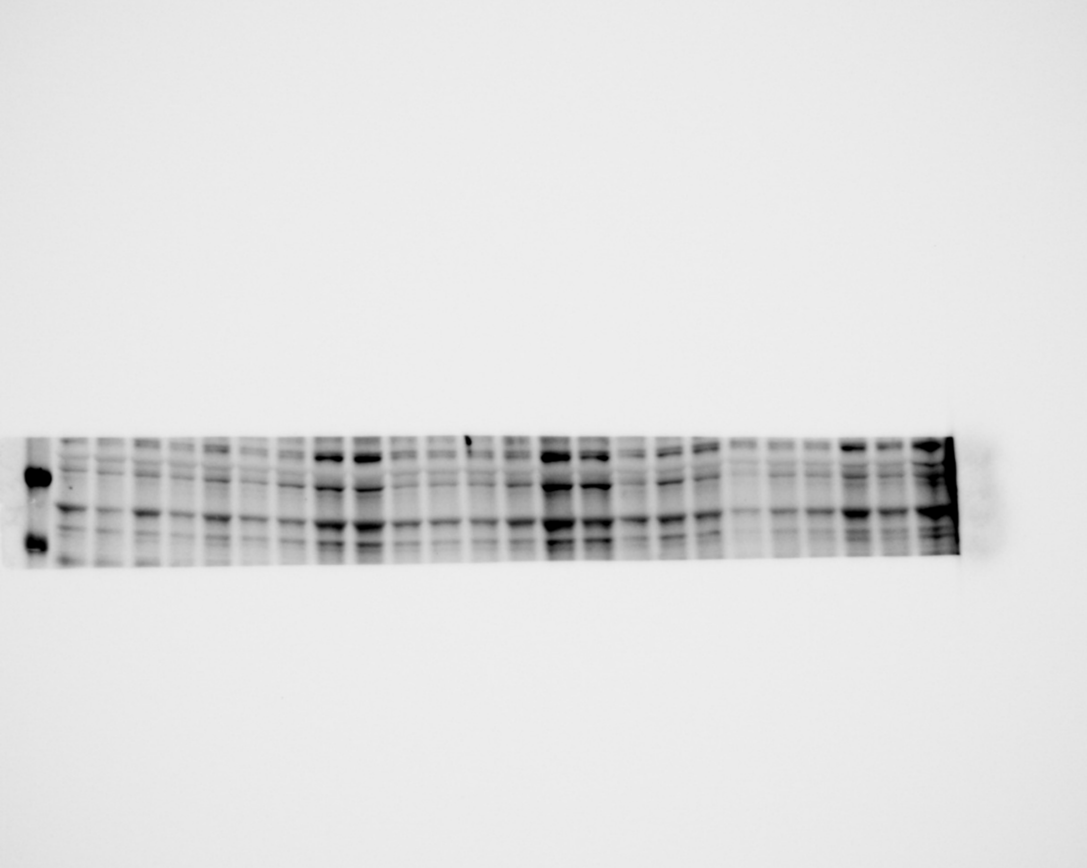

PPARα M3

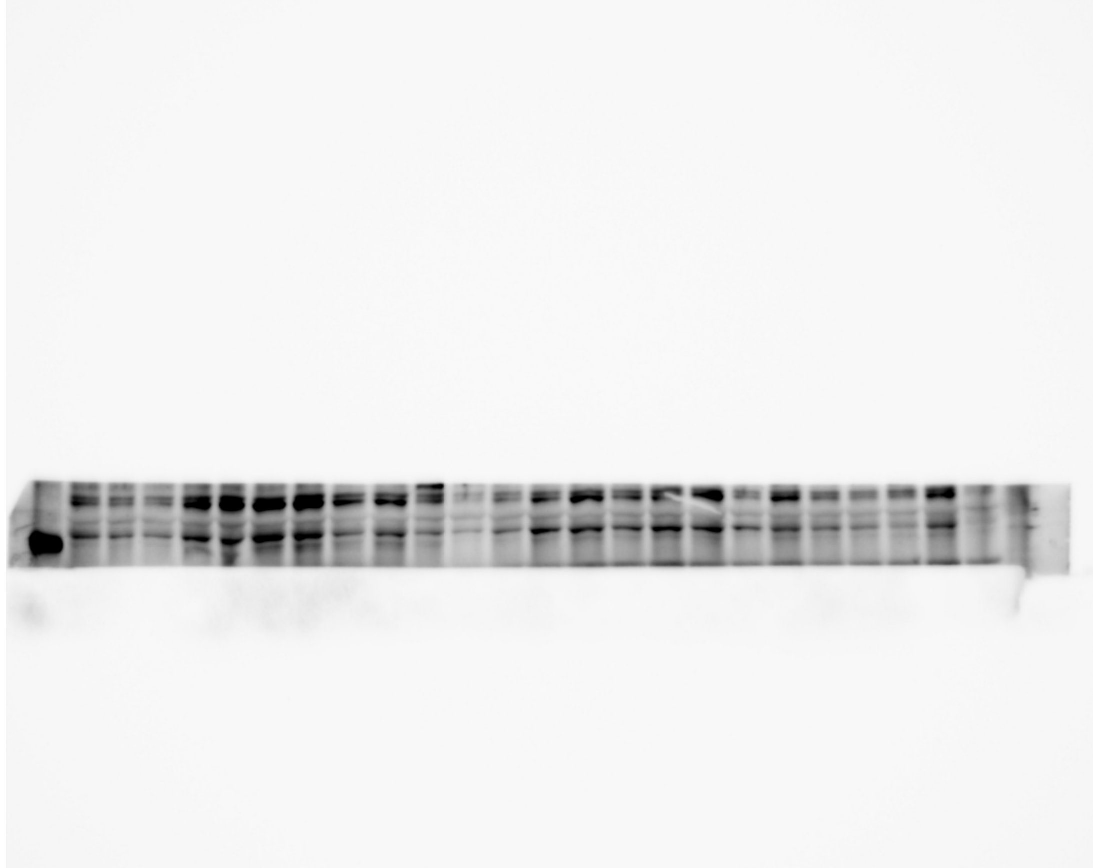

PPARα M3.1

Astrocitos 3xTg

GPR55 (42 kDa)

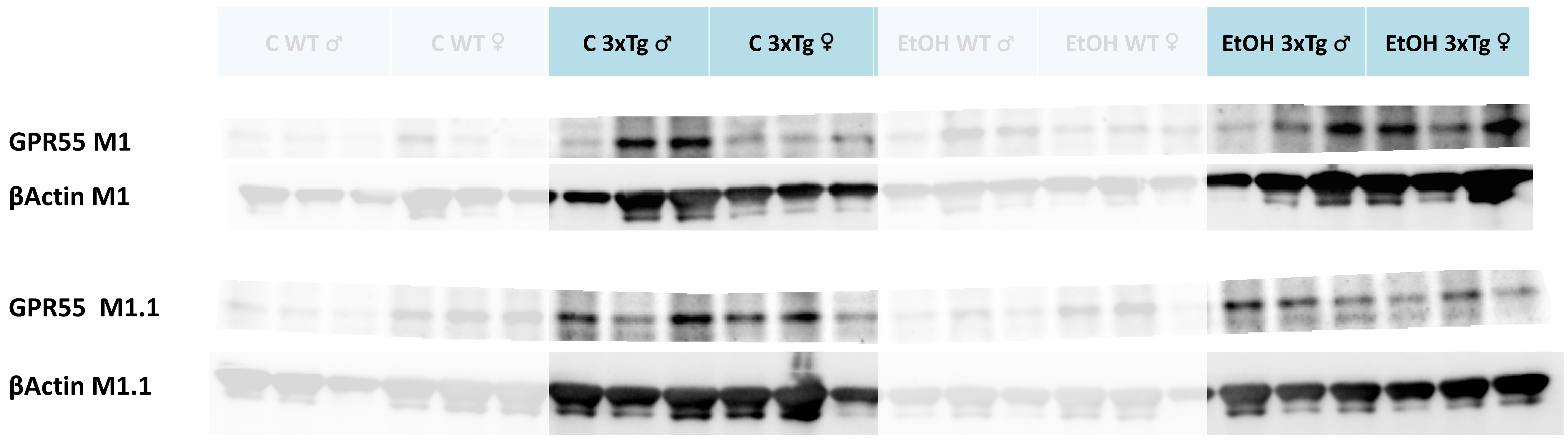

Data for each gel with an n of 3 animals. Both gels have a total n of 6.

UNEDITED BLOTS: GPR55

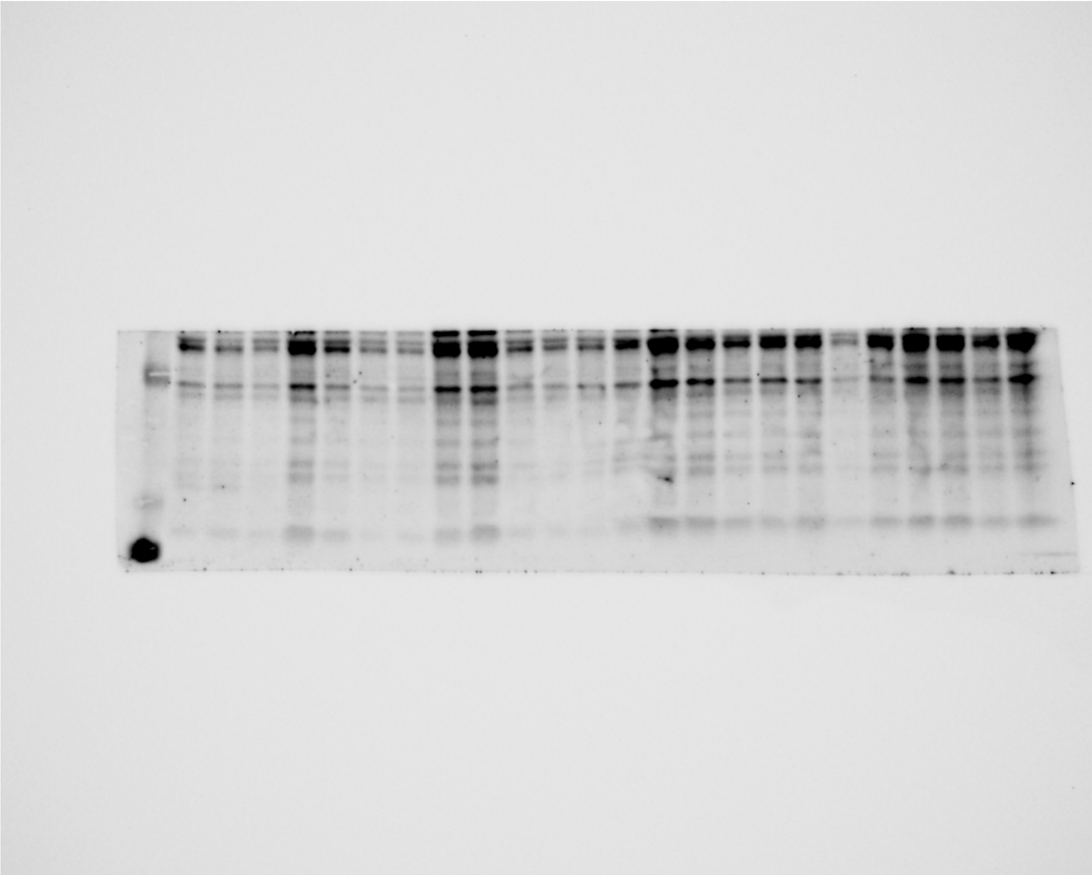

GPR55 M1

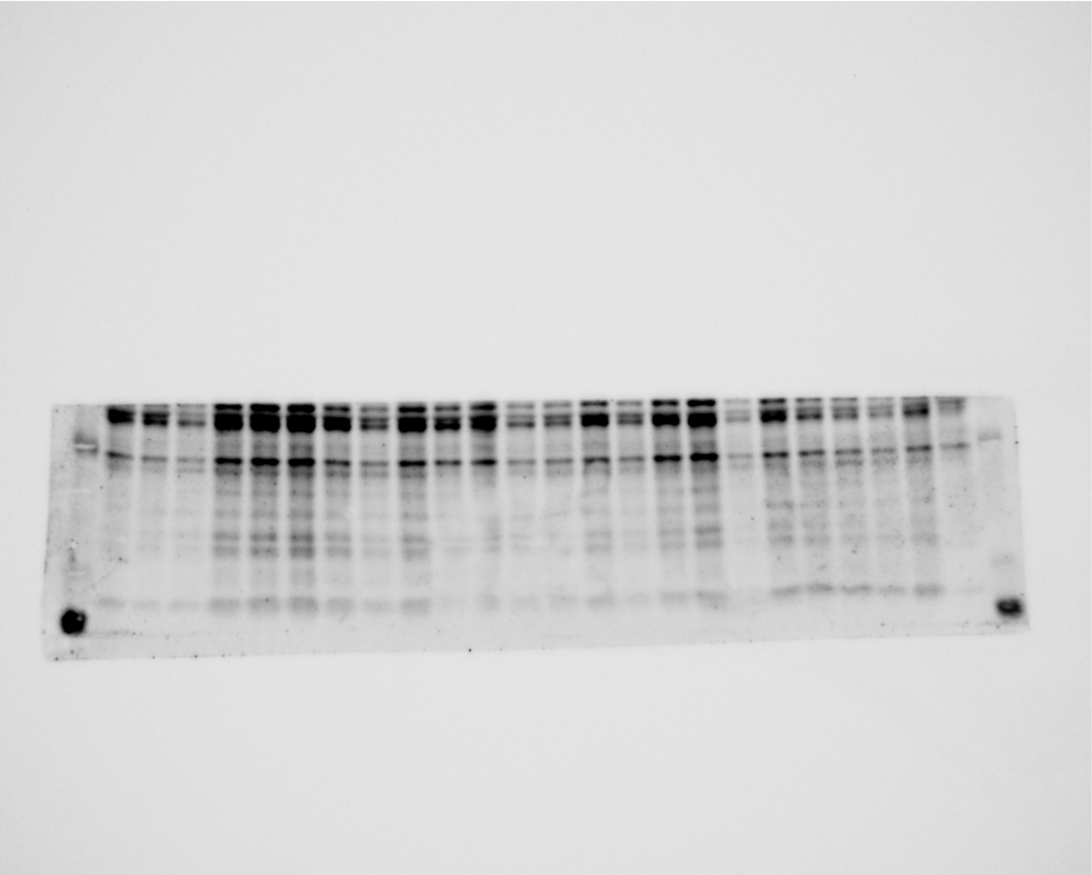

GPR55 M1.1

3xTg astrocytes

NAPE-PLD (46 kDa)

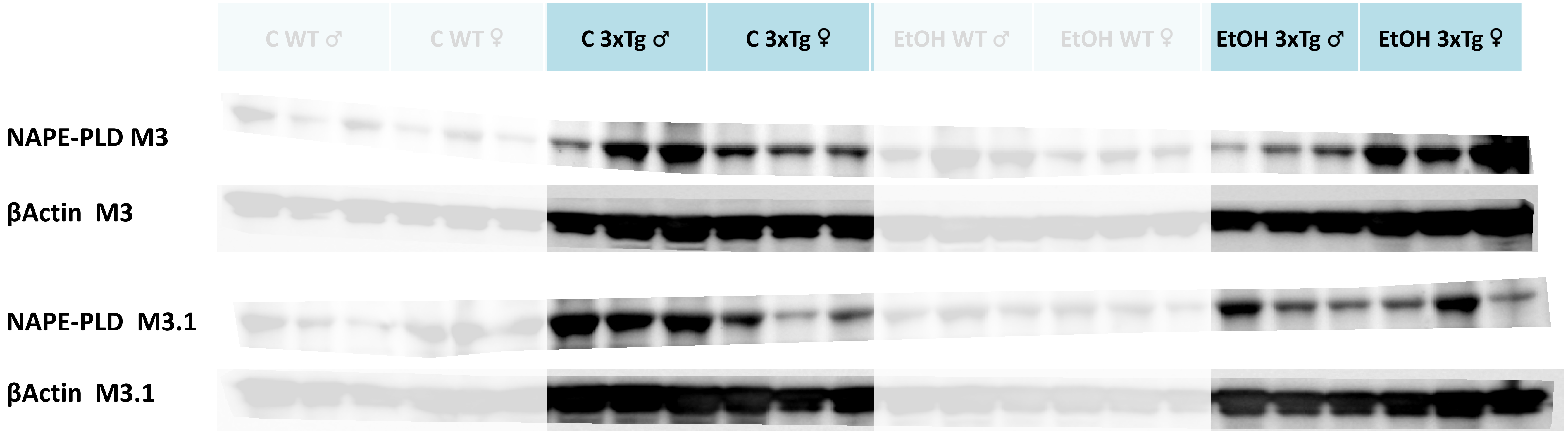

Data for each gel with an n of 3 animals. Both gels have a total n of 6.

UNEDITED BLOTS: NAPE-PLD

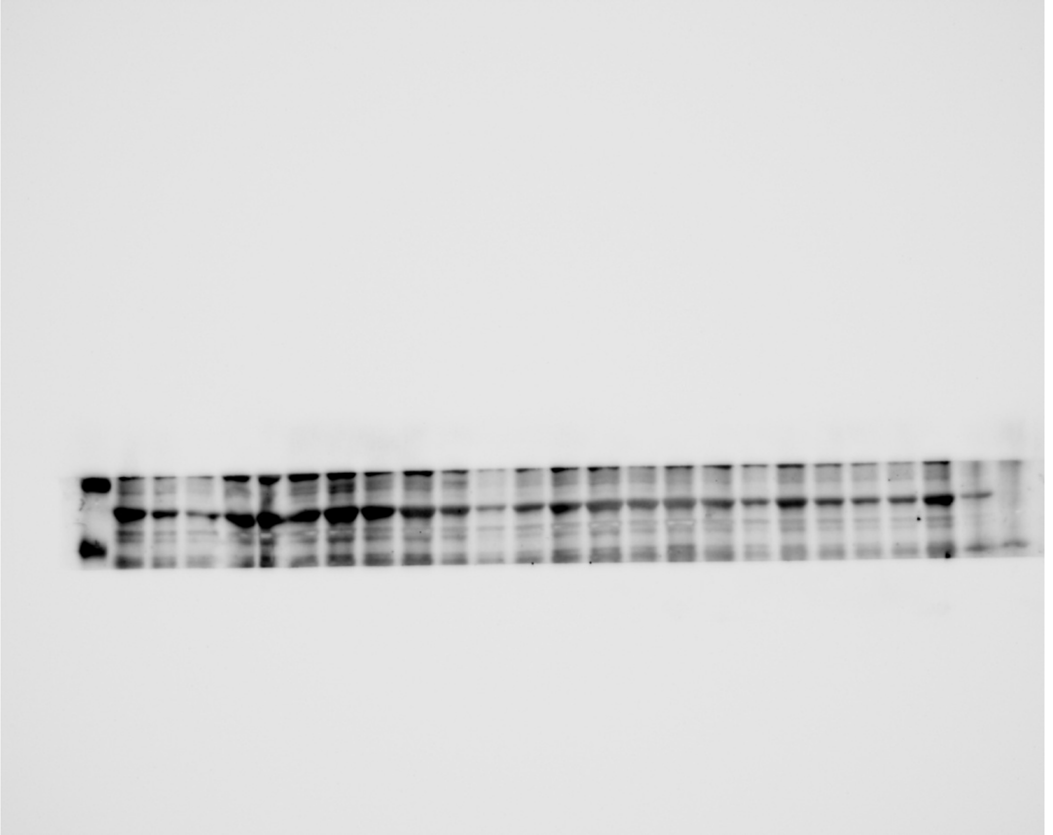

NAPE-PLD M3.1

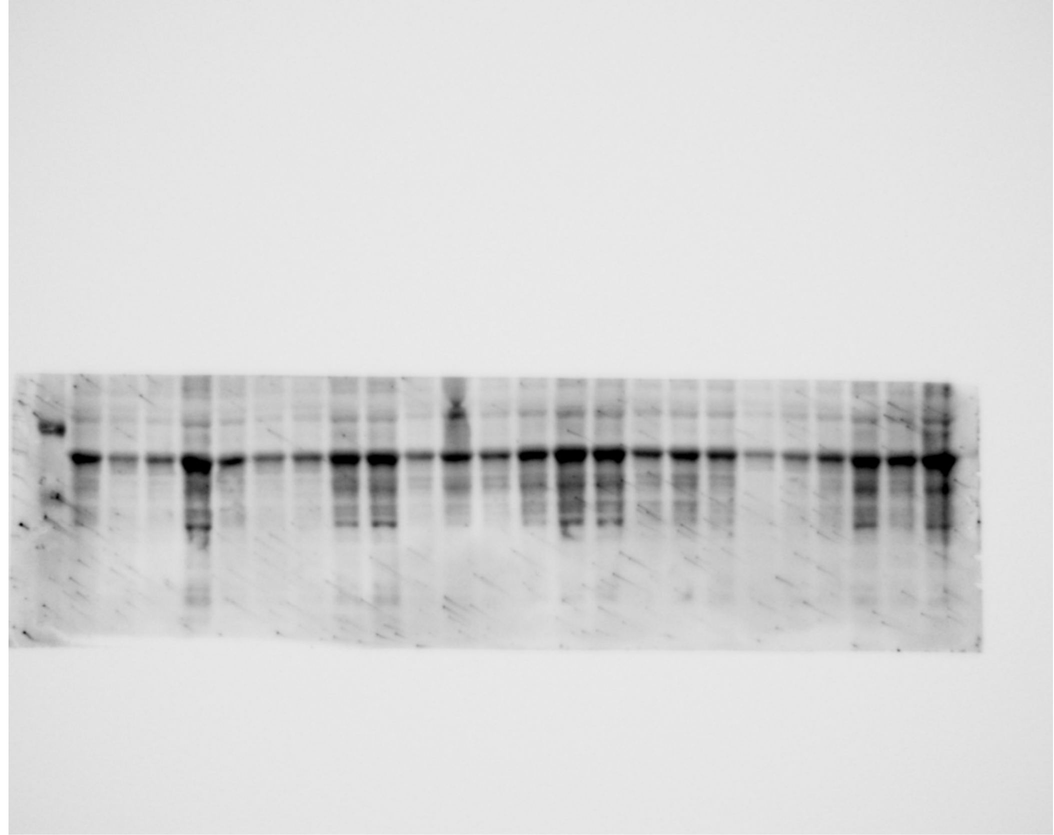

NAPE-PLD M2

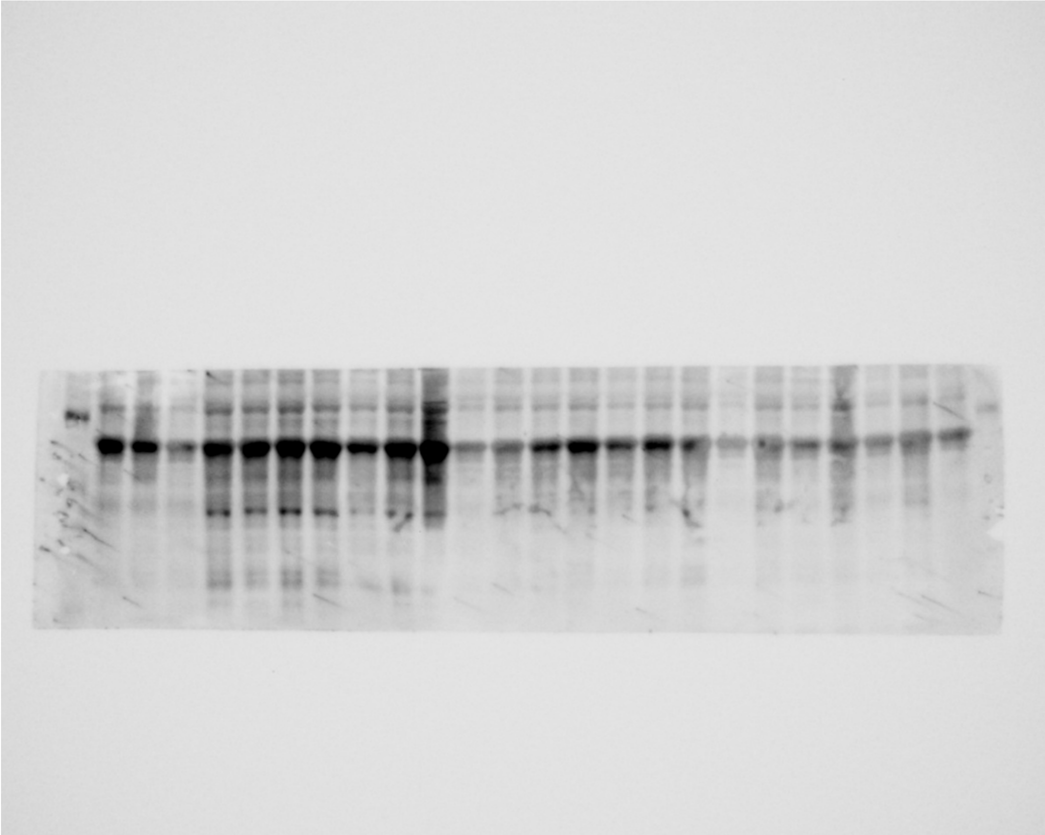

NAPE-PLD M2.1

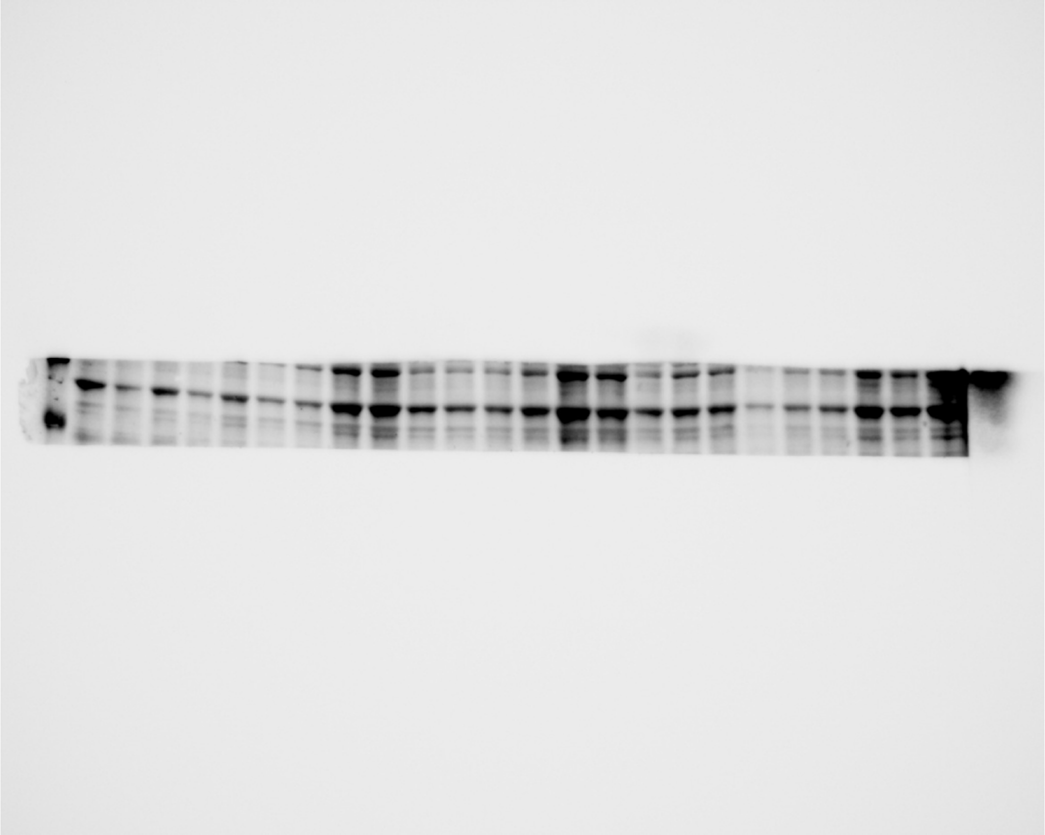

NAPE-PLD M3

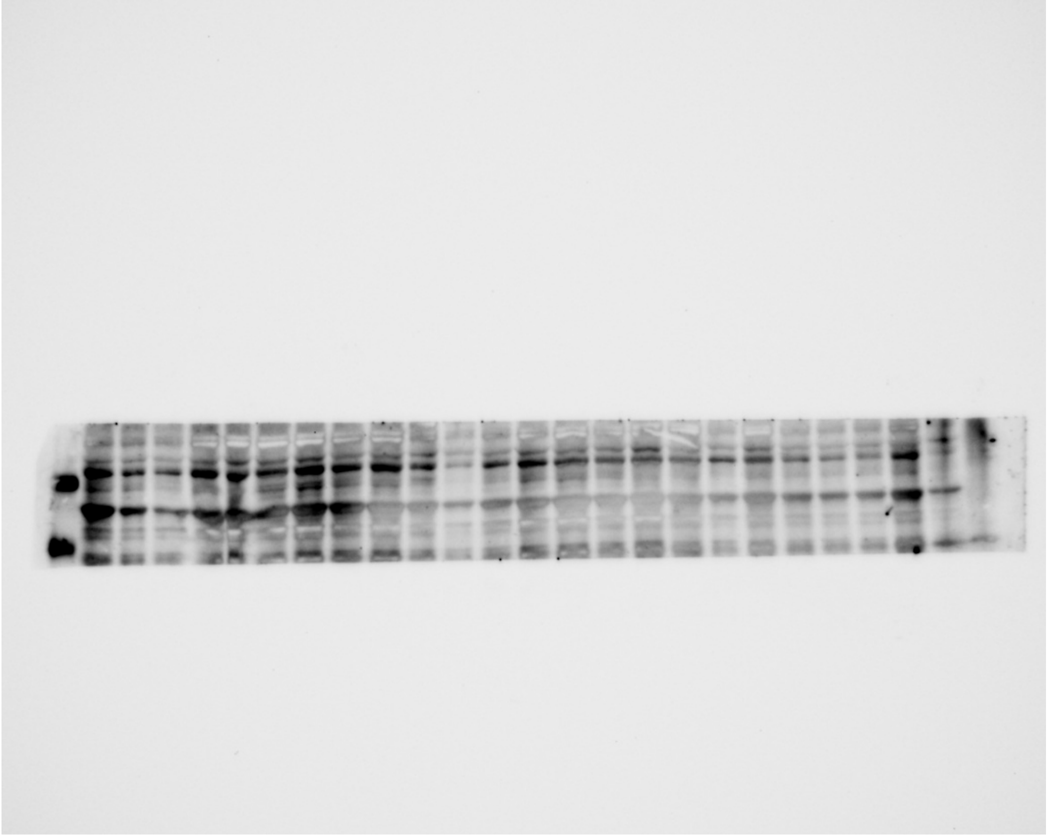

NAPE-PLD M3.1

3xTg astrocytes

MAGL (35 kDa)

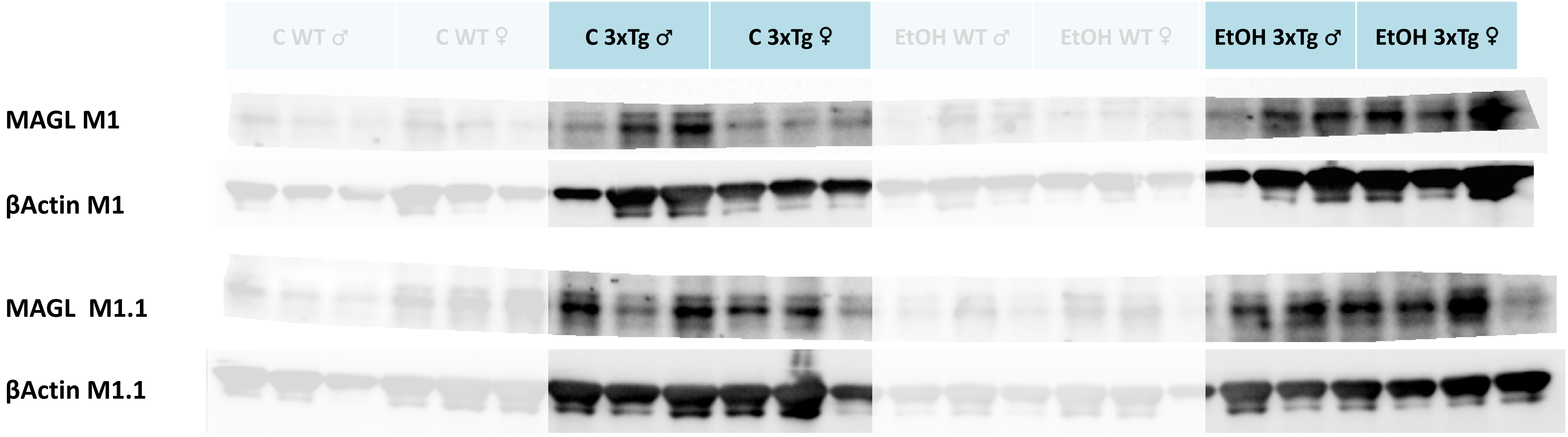

Data for each gel with an n of 3 animals. Both gels have a total n of 6.

UNEDITED BLOTS: MAGL

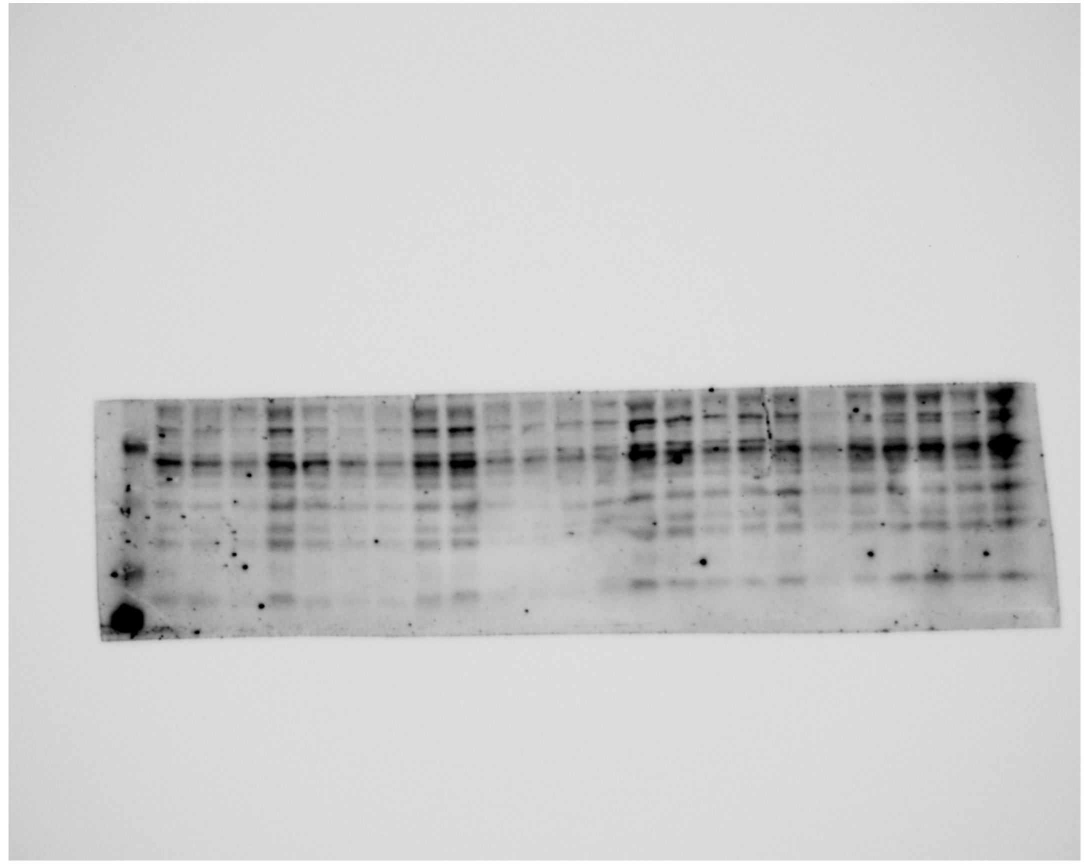

MAGL M1

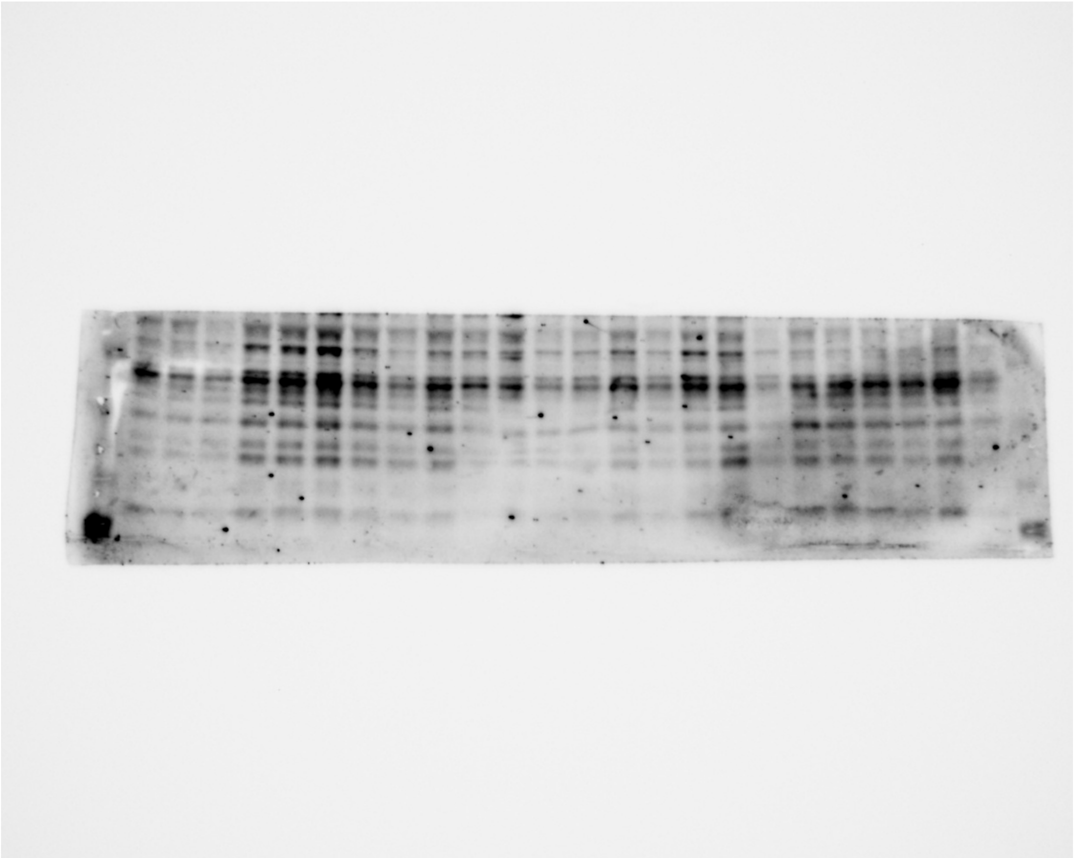

MAGL M1.1

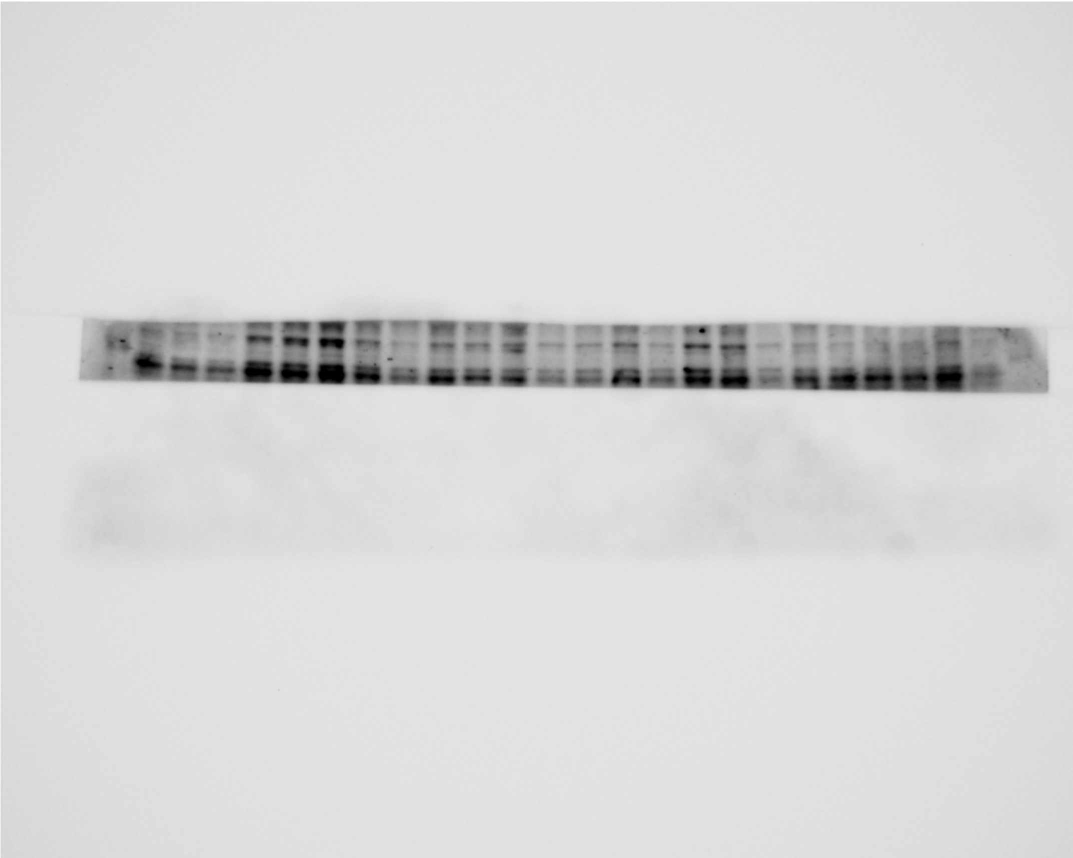

MAGL M1.1

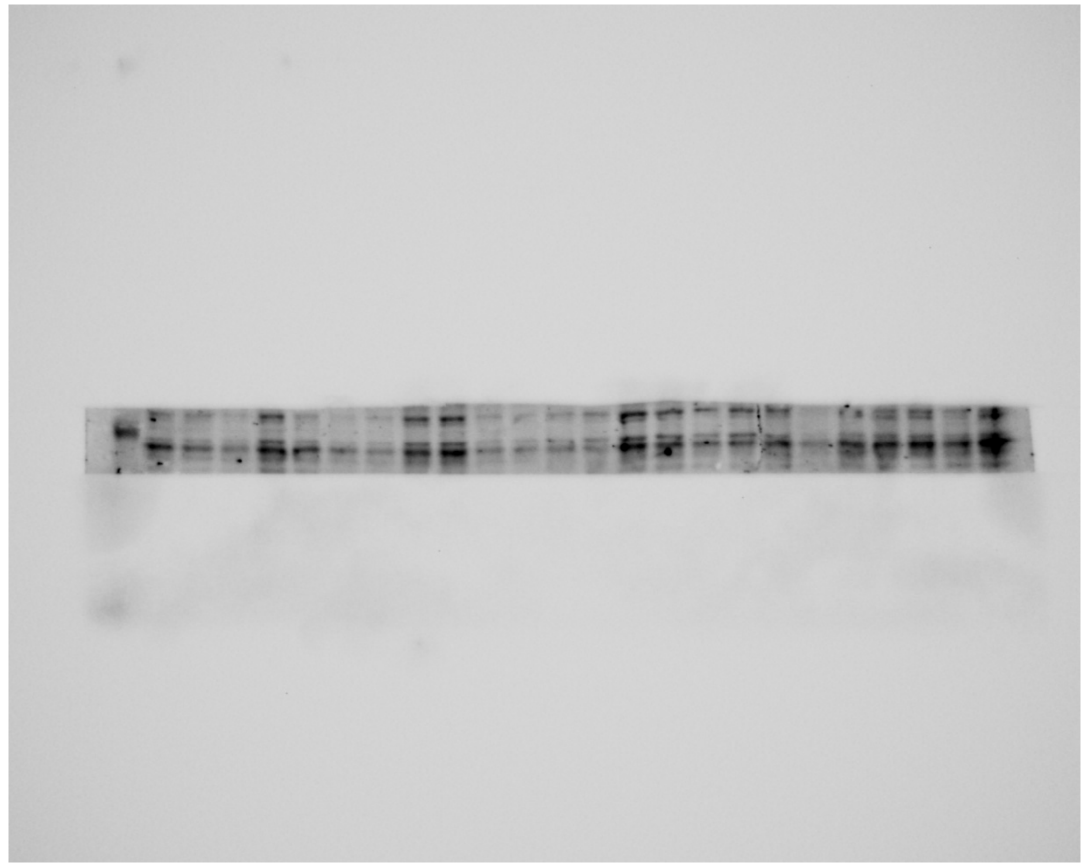

MAGL M1

3xTg astrocytes

FAAH (60 kDa)

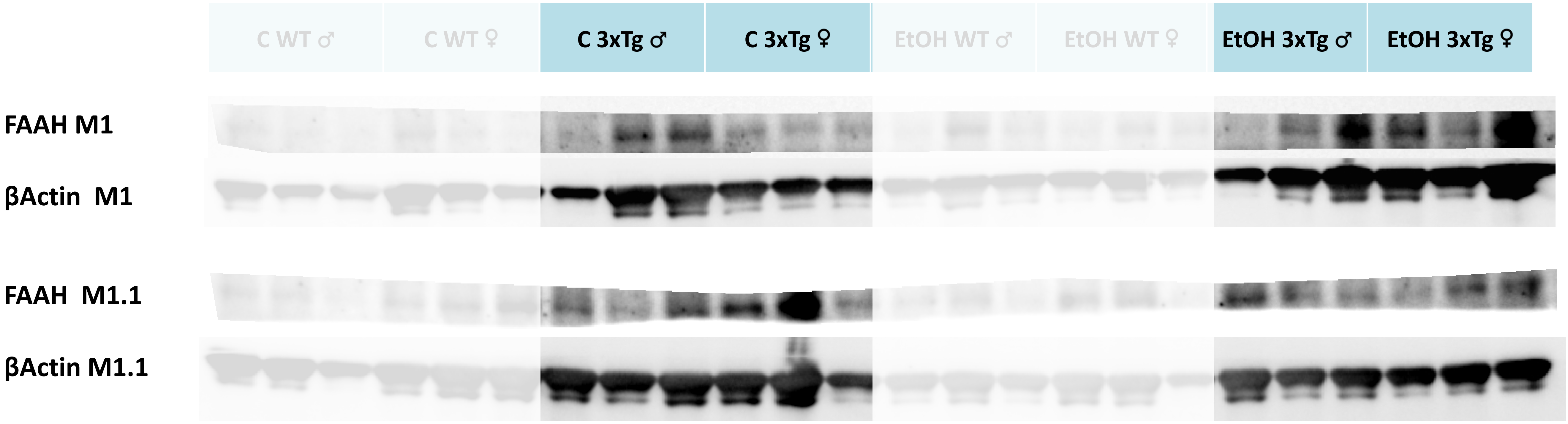

Data for each gel with an n of 3 animals. Both gels have a total n of 6.

UNEDITED BLOTS: FAAH

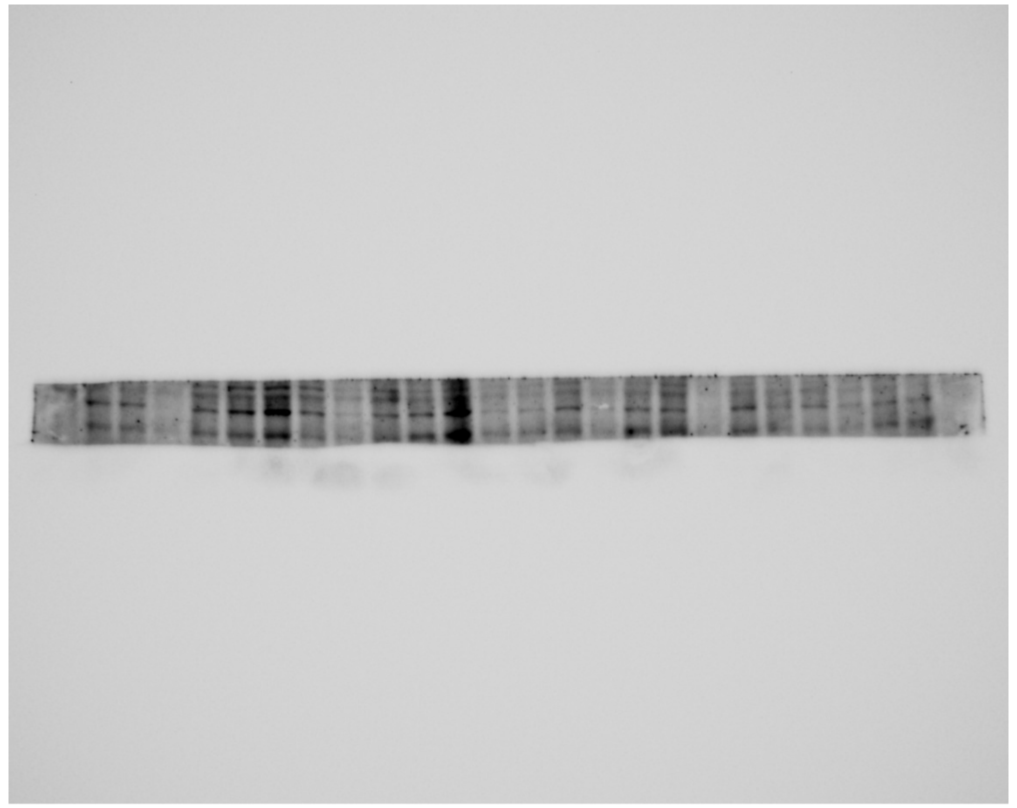

FAAH M1.1

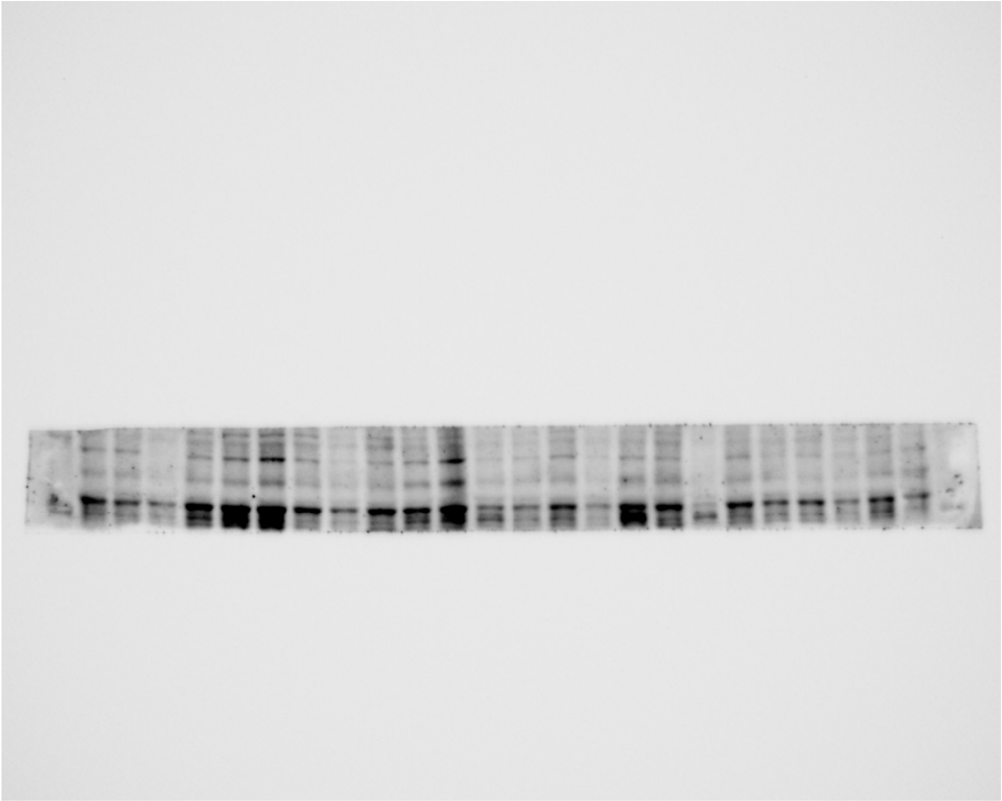

FAAH M1.1

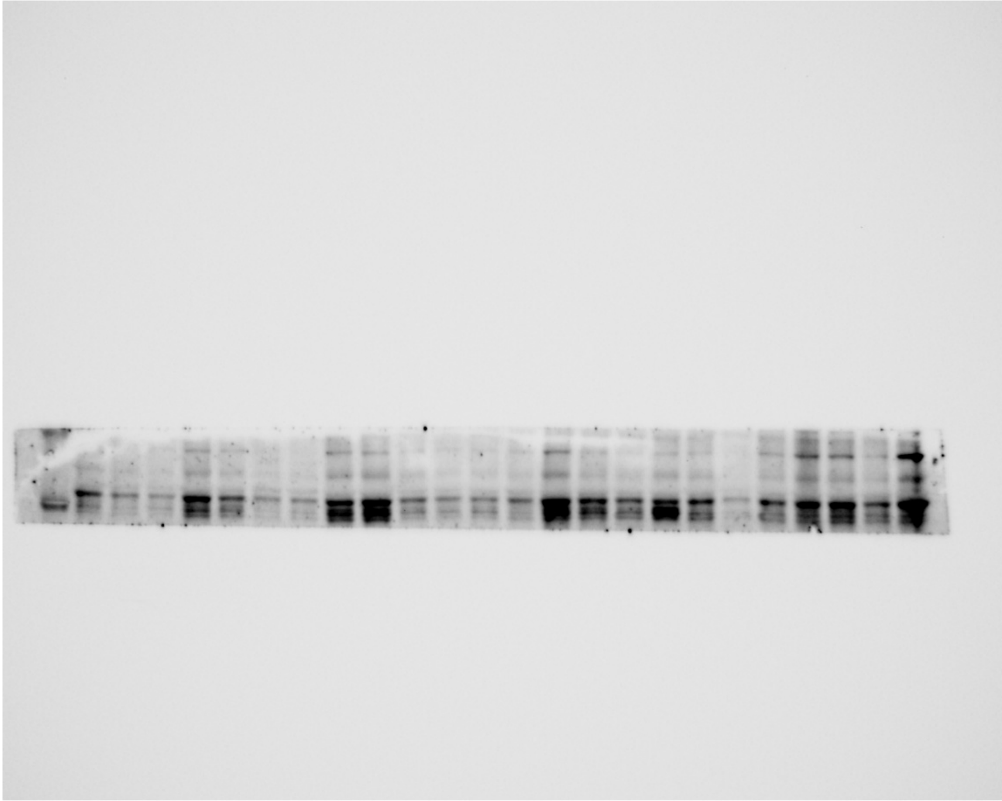

FAAH M1

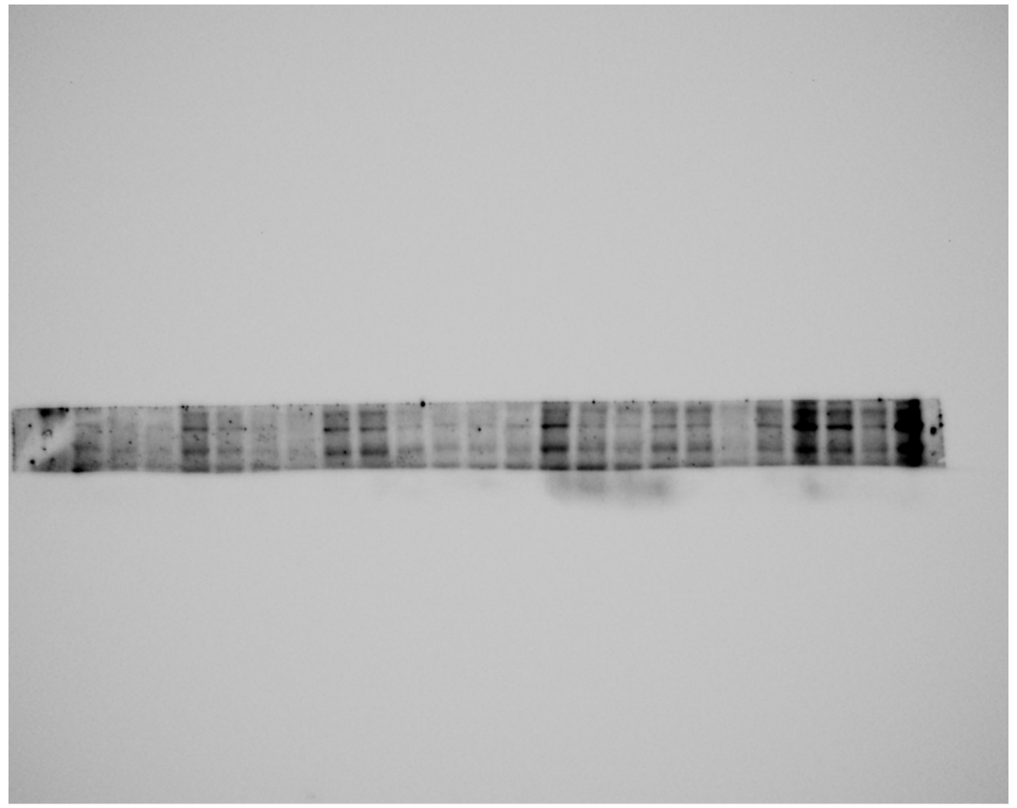

FAAH M1
