# Supplementary material for: Perinatal Ethanol Exposure Induces Astrogliosis and Decreases GRP55/PEA-Mediated Neuroprotection in Hippocampal Astrocytes of the 3×Tg Alzheimer’s Animal Model
Source: Int J Mol Sci. 2025 Nov 18;26(22):11154. doi: 10.3390/ijms262211154 (PMC12652644; doi:10.3390/ijms262211154)
Supplement: Supplementary file 1 [file ijms-26-11154-s001.zip › Table S1. Figures 1 & 7 effect sizes.pdf]

Gfap

Factor	η² Value	Effect Size
Interaction	0.430	**Large**
PEE	0.161	**Large**
Sex	0.063	**Medium**

P2rx5

Factor	η² Value	Effect Size
Interaction	0.373	**Large**
Sex	0.103	**Medium**
PEE	0.000035	**Negligible**

Vimentin

Factor	η² Value	Effect Size
Sex	0.0118	**Small**
PEE	0.0039	**Very Small**
Interaction	0.0035	**Very Small**

Mcu

Factor	η² Value	Effect Size
Sex	0.0773	**Medium**
PEE	0.0431	**Small**
Interaction	0.0042	**Very Small**

Tnfa

Factor	η² Value	Effect Size
PEE	0.2571	**Large**
Sex	0.1405	**Large**
Interaction	0.00043	**Negligible**

Nsmf

Factor	η² Value	Effect Size
Sex	0.1041	**Medium–Large**
PEE	0.00125	**Negligible**
Interaction	0.0000049	**Negligible**

Il6

Factor	η² Value	Effect Size
PEE	0.1032	**Medium**
Sex	0.0319	**Small**
Interaction	0.0131	**Very Small**

Itpr1

Factor	η² Value	Effect Size
Sex	0.0839	**Medium**
Interaction	0.0064	**Very Small**
Treatment	0.0011	**Negligible**

Il1b

Factor	η² Value	Effect Size
Sex	0.200	**Large**
PEE	0.113	**Medium**
Interaction	0.019	**Small**

Gls

Factor	η² Value	Effect Size
Sex	0.4240	**Large**
Interaction	0.0621	**Small–Medium**
PEE	0.0000476	**Negligible**

Ptgs

Factor	η² Value	Effect Size
PEE	0.1153	**Medium**
Sex	0.0128	**Small**
Interaction	0.0005	**Negligible**

Gls2

Factor	η² Value	Effect Size
Treatment	0.0551	**Small–Medium**
Sex	0.0337	**Small**
Interaction	0.0003	**Negligible**
