# Supplementary material for: Perinatal Ethanol Exposure Induces Astrogliosis and Decreases GRP55/PEA-Mediated Neuroprotection in Hippocampal Astrocytes of the 3×Tg Alzheimer’s Animal Model
Source: Int J Mol Sci. 2025 Nov 18;26(22):11154. doi: 10.3390/ijms262211154 (PMC12652644; doi:10.3390/ijms262211154)
Supplement: Supplementary file 1 [file ijms-26-11154-s001.zip › Table S2. Figures 2 & 3 effect sizes.pdf]

Cnr1

Factor	η² Value	Effect Size
Interaction	0.152	**Large**
PEE	0.136	**Large**
Sex	0.010	**Small**

Cnr2

Factor	η² Value	Effect Size
Interaction	0.103	**Medium**
PEE	0.0096	**Small**
Sex	0.0045	**Very Small**

Gpr55

Factor	η² Value	Effect Size
PEE	0.294	**Large**
Sex	0.0088	**Small**
Interaction	0.0014	**Very Small**

Ppara

Factor	η² Value	Effect Size
Sex	0.4165	**Large**
PEE	0.00156	**Very Small**
Interaction	0.00347	**Very Small**

Trpv1

Factor	η² Value	Effect Size
Interaction	0.0573	**Medium**
PEE	0.0167	**Small**
Sex	0.00026	**Negligible**

Dagla

Factor	η² Value	Effect Size
Interaction	0.0933	**Medium**
PEE	0.067	**Medium**
Sex	0.0053	**Very Small**

Daglb

Factor	η² Value	Effect Size
Interaction	0.2446	**Large**
PEE	0.0774	**Medium**
Sex	0.0195	**Small**

Mgll

Factor	η² Value	Effect Size
PEE	0.355	**Large**
Interaction	0.0288	**Small**
Sex	0.00003	**Negligible**

Nape-pld

Factor	η² Value	Effect Size
Interaction	0.0735	**Medium**
PEE	0.0729	**Medium**
Sex	0.0120	**Small**

Faah

Factor	η² Value	Effect Size
PEE	0.473	**Large**
Interaction	0.0032	**Very Small**
Sex	0.0029	**Very Small**

Napepld/Faah

Factor	η² Value	Effect Size
PEE	0.299	**Large**
Interaction	0.136	**Large**
Sex	0.083	**Medium**

Dagla/Mgll

Factor	η² Value	Effect Size
Sex	0.0276	**Small**
PEE	0.0156	**Small**
Interaction	0.0021	**Very Small**

Daglb/Mgll

**Factor**	**η^2 Value**	**Effect Size**
Interaction	0.2448	**Large**
Treatment	0.0775	**Medium**
Sex	0.0195	**Small**
