# Supplementary material for: Perinatal Ethanol Exposure Induces Astrogliosis and Decreases GRP55/PEA-Mediated Neuroprotection in Hippocampal Astrocytes of the 3×Tg Alzheimer’s Animal Model
Source: Int J Mol Sci. 2025 Nov 18;26(22):11154. doi: 10.3390/ijms262211154 (PMC12652644; doi:10.3390/ijms262211154)
Supplement: Supplementary file 1 [file ijms-26-11154-s001.zip › Table S3. Figures 4 & 5 effect sizes.pdf]

CB1

Factor	η^2 Value	Effect Size
Interaction	0.0127	**Small**
PEE	0.0117	**Small**
Sex	0.0016	**Negligible**

CB2

Factor	η^2 Value	Effect Size
Interaction	0.2636	**Large**
PEE	0.1104	**Medium**
Sex	0.0888	**Medium**

PPARα

Factor	η^2 Value	Effect Size
Sex	0.245	**Large**
Interaction	0.145	**Medium–Large**
PEE	0.036	**Small**

GPR55

Factor	η^2 Value	Effect Size
Sex	0.2139	**Large**
PEE	0.0495	**Small–Medium**
Interaction	0.00004	**Negligible**

DAGLα

Factor	η^2 Value	Effect Size
PEE	0.3641	**Large**
Interaction	0.1615	**Medium–Large**
Sex	0.0082	**Very Small**

DAGLβ

Factor	η^2 Value	Effect Size
Interaction	0.0961	**Medium**
PEE	0.0176	**Small**
Sex	0.0105	**Small**

MAGL

Factor	η^2 Value	Effect Size
PEE	0.1887	**Large**
Sex	0.0861	**Medium**
Interaction	0.0006	**Negligible**

NAPE-PLD

Factor	η^2 Value	Effect Size
Interaction	0.3129	**Large**
PEE	0.0015	**Negligible**
Sex	0.0004	**Negligible**

FAAH

Factor	η^2 Value	Effect Size
Sex	0.0162	**Small**
PEE	0.0151	**Small**
Interaction	0.000007	**Negligible**

DAGLα/MAGL

Factor	η^2 Value	Effect Size
PEE	0.3223	**Large**
Interaction	0.1313	**Medium–Large**
Sex	0.0855	**Medium**

DAGLβ/MAGL

Factor	η^2 Value	Effect Size
PEE	0.0299	**Small**
Sex	0.0177	**Small**
Interaction	0.0169	**Small**

NAPE-PLD/FAAH

Factor	η^2 Value	Effect Size
Interaction	0.2182	**Large**
PEE	0.0355	**Small**
Sex	0.00002	**Negligible**
