# Supplementary material for: Perinatal Ethanol Exposure Induces Astrogliosis and Decreases GRP55/PEA-Mediated Neuroprotection in Hippocampal Astrocytes of the 3×Tg Alzheimer’s Animal Model
Source: Int J Mol Sci. 2025 Nov 18;26(22):11154. doi: 10.3390/ijms262211154 (PMC12652644; doi:10.3390/ijms262211154)
Supplement: Supplementary file 1 [file ijms-26-11154-s001.zip › Table S4. Figure 6 effect sizes.pdf]

2AG

Factor	η^2 Value	Effect Size
Sex	0.1477	**Large**
PEE	0.0814	**Medium**
Interaction	0.0320	**Small**

2LG

Factor	η^2 Value	Effect Size
PEE	0.0507	**Small–Medium**
Interaction	0.0247	**Small**
Sex	0.0002	**Negligible**

POEA

Factor	η^2 Value	Effect Size
Sex	0.0749	**Medium**
PEE	0.0576	**Small–Medium**
Interaction	0.0043	**Negligible**

LEA

Factor	η^2 Value	Effect Size
PEE	0.1105	**Medium**
Interaction	0.0027	**Negligible**
Sex	0.0027	**Negligible**

PEA

Factor	η^2 Value	Effect Size
PEE	0.2908	**Large**
Interaction	0.0285	**Small**
Sex	0.0003	**Negligible**

OEA

Factor	η^2 Value	Effect Size
PEE	0.0324	**Small**
Interaction	0.0321	**Small**
Sex	0.0004	**Negligible**

SEA

Factor	η^2 Value	Effect Size
PEE	0.0485	**Small–Medium**
Interaction	0.0393	**Small**
Sex	0.0169	**Small**
