# Supplementary material for: Perinatal Ethanol Exposure Induces Astrogliosis and Decreases GRP55/PEA-Mediated Neuroprotection in Hippocampal Astrocytes of the 3×Tg Alzheimer’s Animal Model
Source: Int J Mol Sci. 2025 Nov 18;26(22):11154. doi: 10.3390/ijms262211154 (PMC12652644; doi:10.3390/ijms262211154)
Supplement: Supplementary file 1 [file ijms-26-11154-s001.zip › Table S5. Degrees of freedom.pdf]

Variable analyzed	Initial total N	N per group	Outliers	Final total N	Df Error (N-4)	F reported
Cnr1	35	8-9	2	33	29	F (1, 29)
Cnr2	35	8-9	1	34	30	F (1, 30)
Gpr55	35	8-9	3	32	28	F (1, 28)
Ppara	24	6	0	24	20	F (1, 20)
Trpv1	23	5-6	0	23	19	F (1, 19)
Dagla	35	8-9	1	34	30	F (1, 30)
Daglb	35	8-9	0	35	31	F (1, 31)
Mgll	35	8-9	3	32	28	F (1, 28)
Nape-pld	35	8-9	0	35	31	F (1, 31)
Faah	35	8-9	4	31	27	F (1, 27)
Dagla/Mgll	35	8-9	1	34	30	F (1, 30)
Daglb/Mgll	35	8-9	0	35	31	F (1, 31)
Napepld/Faah	35	8-9	3	32	28	F (1, 28)
CB1	24	6	0	24	20	F (1, 20)
CB2	24	6	0	24	20	F (1, 20)
PPARa	24	6	0	24	20	F (1, 20)
GPR55	24	6	0	24	20	F (1, 20)
DAGLa	24	6	0	24	20	F (1, 20)
DAGLb	24	6	0	24	20	F (1, 20)
MAGL	24	6	0	24	20	F (1, 20)
NAPE-PLD	24	6	0	24	20	F (1, 20)
FAAH	24	6	0	24	20	F (1, 20)
DAGLA/MGLL	24	6	0	24	20	F (1, 20)
DAGLB/MGLL	24	6	0	24	20	F (1, 20)
NAPEPLD/FAAH	24	6	0	24	20	F (1, 20)
2AG	20	5	3	17	13	F (1, 13)
2LG	13	3-4	0	13	9	F (1, 9)
POEA	15	3-4	0	15	11	F (1, 11)
LEA	20	5	3	17	13	F (1, 13)
PEA	18	4-5	0	18	14	F (1, 14)
OEA	20	5	2	18	14	F (1, 14)
SEA	19	4-5	3	16	12	F (1, 12)
P2rx5	24	6	0	24	20	F (1, 20)
Mcu	24	6	3	21	17	F (1, 17)
Nsmf	24	6	0	24	20	F (1, 20)
Itpr1	24	6	1	23	19	F (1, 19)
Gls	24	6	0	24	20	F (1, 20)
Gls2	24	6	1	23	19	F (1, 19)
